# Glyphosate Separating and Sensing for Precision Agriculture and Environmental Protection in the Era of Smart Materials

**DOI:** 10.1021/acs.est.3c01269

**Published:** 2023-06-29

**Authors:** Jarosław Mazuryk, Katarzyna Klepacka, Włodzimierz Kutner, Piyush Sindhu Sharma

**Affiliations:** † Department of Electrode Processes, Institute of Physical Chemistry, Polish Academy of Sciences, 01-224 Warsaw, Poland; ‡ Bio & Soft Matter, Institute of Condensed Matter and Nanosciences, Université catholique de Louvain, 1 Place Louis Pasteur, 1348 Louvain-la-Neuve, Belgium; § Functional Polymers Research Team, Institute of Physical Chemistry, Polish Academy of Sciences, 01-224 Warsaw, Poland; ∥ ENSEMBLE3 sp. z o. o., 01-919 Warsaw, Poland; ⊥ Faculty of Mathematics and Natural Sciences. School of Sciences, Cardinal Stefan Wyszynski University in Warsaw, 01-938 Warsaw, Poland; # Modified Electrodes for Potential Application in Sensors and Cells Research Team, Institute of Physical Chemistry, Polish Academy of Sciences, 01-224 Warsaw, Poland

**Keywords:** engineered nanomaterials, environmental pollution, glyphosate-based herbicides, lab-on-a-chip, precision agriculture, smart material-based sensors

## Abstract

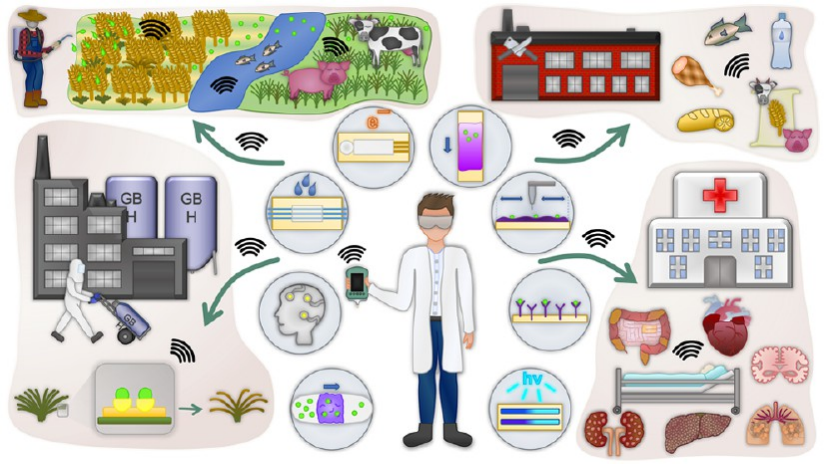

The present article critically and comprehensively reviews the most recent reports on smart sensors for determining glyphosate (GLP), an active agent of GLP-based herbicides (GBHs) traditionally used in agriculture over the past decades. Commercialized in 1974, GBHs have now reached 350 million hectares of crops in over 140 countries with an annual turnover of 11 billion USD worldwide. However, rolling exploitation of GLP and GBHs in the last decades has led to environmental pollution, animal intoxication, bacterial resistance, and sustained occupational exposure of the herbicide of farm and companies’ workers. Intoxication with these herbicides dysregulates the microbiome-gut-brain axis, cholinergic neurotransmission, and endocrine system, causing paralytic ileus, hyperkalemia, oliguria, pulmonary edema, and cardiogenic shock. Precision agriculture, i.e., an (information technology)-enhanced approach to crop management, including a site-specific determination of agrochemicals, derives from the benefits of smart materials (SMs), data science, and nanosensors. Those typically feature fluorescent molecularly imprinted polymers or immunochemical aptamer artificial receptors integrated with electrochemical transducers. Fabricated as portable or wearable lab-on-chips, smartphones, and soft robotics and connected with SM-based devices that provide machine learning algorithms and online databases, they integrate, process, analyze, and interpret massive amounts of spatiotemporal data in a user-friendly and decision-making manner. Exploited for the ultrasensitive determination of toxins, including GLP, they will become practical tools in farmlands and point-of-care testing. Expectedly, smart sensors can be used for personalized diagnostics, real-time water, food, soil, and air quality monitoring, site-specific herbicide management, and crop control.

## 1 Smart Materials-Based Sensors in Precision Agriculture

### 1.1 Ecological Contamination with Glyphosate

According to the United Nations Food and Agriculture Organization (FAO), the world’s population will attain ∼9.7 billion by 2050, corresponding to a 32% projected growth.^1^ A recent meta-analysis of projected global food demand revealed that the total global food demand should increase by 35% to 56% between 2010 and 2050.^2^ The food shortage threat belongs to global agriculture’s most harmful socio-economic and environmental challenges.^3^ Aside from the COVID-19 pandemic,^4^ increases in temperature and atmospheric CO_2_ concentration, the environmental pollution of agrochemicals arising from ill-considered farming and insufficient fertilizer delivery systems has become a civilization problem.^5^ 2020-Forecasted global agrochemical annual use was 120 million tons for nitrogen-based fertilizers, 50 million tons for phosphate-based fertilizers, and over 2.6 million tons for pesticides.^6,7^ In recent decades, the nutrient-use efficiency (NUE) has dropped significantly, i.e., over 50% of the N, 85% of the P are not assimilated by crops,^8,9^ and less than 10% of the applied pesticides reach their targets.^5^ If not handled, these usages were estimated to increase by 50–90% by 2050.^5,10^

The first global initiative to solve agriculture’s challenges began with the Third Agricultural (Green) Revolution in the 1950s and 1960s.^11^ This technology transfer involved (i) high-throughput cultivation of high-yielding varieties of cereal seeds, (ii) improvement of the NUE by spatiotemporal biofertilization and microbial biodiversity, and (iii) reduction of the reactive nitrogen species use and nitrogen oxide emission. In the 1970s, it was followed by the Gene Revolution,^12^ based on the extensive use of genetically engineered (GE) herbicide-resistant crops, especially glyphosate (GLP)-resistant plants, which resulted in yield increases, tillage reduction, and enhanced the high technology-based weed management.^13^ Yet, despite several advantages of lowering greenhouse gas emission,^14^ the large-scale misuse of GLP-based herbicides (GBHs) and GLP-resistant crops and GLP-resistant weed-originated single mode-of-action herbicides has caused environmental pollution with GLP, herbicide resistance, superweeds, and pests generation, as well as consuming GE organisms and GBH-contaminated products, which have consequently re-empowered the expensive tillage.^13,15^ These outcomes have boosted the international debate on the policy controlling or forbidding GBH exploitation and, on the other hand, developing sensors for GLP contaminants that emerged in the environment over the last 50 years.^16^ Emerging malnourishment, inappropriate weed management, and critically imbalanced and anthropogenically altered P-cycle have been recognized by the U.S. National Science Foundation as some of the most crucial challenges of modern and future agriculture and ecology, which require advances both in proper fertilizing and devising portable and sensitive tools of chemical analysis in agriculture and ecology.^3,17,18^

### 1.2 Smart Materials in Precision Agriculture

The latest advancements in agriculture and ecology originated with precision agriculture (PA). According to the International Society of Precision Agriculture (ISPAg) (https://www.ispag.org),^19^ PA is “a management strategy that gathers, processes, and analyzes temporal, spatial, and individual data and combines them with other information to support management decisions according to estimated variability for improved resource use efficiency, productivity, quality, profitability and sustainability of agricultural production.” PA’s most crucial challenge is reducing herbicide resistance in weed management. In 2021, the herbicide market value accounted for ∼30 billion USD and was forecasted to reach ∼40 billion USD by 2027 (https://www.imarcgroup.com/herbicides-market). Expectedly, herbicide-resistant weed management (HRWM) shall comprise almost half of the modern agricultural activities, including tillage, crop and herbicide diversity, and growing GE herbicide-resistant plants.^20^ The increase in the NUE and crop productions will be achieved by applying artificial intelligence (AI)-excelled devices and smart materials (SMs),^21^ including engineered nanomaterials (ENMs) and biomaterials,^22^ and plant wearable (PW) sensors, actuators, and soft robotics.^23^ These PA HRWM nanobiotechnological tools enable direct, spatiotemporally targeted, and dose-dependent delivery as well as rapid, ultrasensitive, and selective determination of herbicides.

SMs are well-defined, self-sensing, self-healing, and stimuli-responsive materials that change their properties and act according to their surroundings or external stimuli, including microorganisms, chemical compounds, heat, pH, temperature, electromagnetic field, light, humidity, ultrasounds, pressure, and mechanical factors.^21^ The SMs are mostly based on electroactive, piezoelectric, shape memory, and biocompatible polymers. They are integrated with electronic devices implanted or installed in the site of interest.^21,23^ Moreover, these devices, e.g., lab-on-chips (LOCs), microrobots, or smartphone-assisted nanosensors, exploit incorporated computational algorithms that allow for acquiring, processing, and analyzing vast amounts of data in real-time, thus providing a model simulation of spatial and seasonal distribution as well as risk assessment of agrochemicals.^3,17,18,24^

Regarding PA-dedicated SM sensors, there is a need for advanced inexpensive, highly efficient, multifunctional, and flexible consumer- and operator-friendly tools that can determine analytes, including toxins, pests, herbicides, and microbes in nonlaboratory settings, hardly accessible locations, and diverse agroecosystems.^25,26^ Conventional multimodal chemosensors utilize immunochemical receptors, targeted to toxic fertilizers and pesticide residuals, and electrochemical and/or optical transducers. However, because of the environmental systems’ complexity, vulnerabilities, and uncertainties, these conventional sensors will hopefully be upgraded or replaced with smart sensors equipped with computational devices.^27^ In AI PA smart sensors, these conventional sensors are assembled or virtually connected with computational devices that convey the machine learning (ML), deep learning (DL), artificial neural networks, nanoinformatics, and translational bioinformatics algorithms to integrate, compute, process, analyze, and interpret the massive amounts of spatiotemporal data.^17,3,18^ Hence, PA SM sensors, based on smartphones, soft robotics, robotic-automated vehicles, and drones, provide rapid, mobile, and high-throughput analysis. Moreover, they afford high-quality, decision-making outcomes for herbicide misuse control and sustained and profitable agricultural production and weed management.^3,17,18,28,29^ For instance, DL-enhanced computing methods enable the analysis of high-resolution spectral images of herbicide-sprayed plants mapped by scanning transmission X-ray microscopy or X-ray fluorescence spectroscopy.^30,31^

Data science-excelled and PA-targeted SM sensors can generally analyze short- or long-term weather conditions, soil properties, plant diseases, pests, microbial communities, and industrial pollution.^3,17,18^ Smart sensors and sensor networks must sense and sustain optimal conditions for plant cultivation, including moisture, temperature, pH, nutrients, and agrochemicals.^3^ Environment-friendly AI PA targets the dynamic and complex nature of the local agroecosystem by synergistic application of theoretical prediction models and experimental stimuli-responsive delivery-detection tools. If attached or administrated to the soil, plant, or crops, the AI PA sensor can capture, digitize, and process images, as well as detect, monitor, respond, and regulate physicochemical stimuli and atmospheric conditions in an information-supported decision-making manner.^18^ Since pests cause 34% of crop loss globally, controlling and increasing crop yields is essential.^32^ By predicting the ecosystem components’ behavior, PA provides a real-time response to weather conditions, nutrient cycling, crop growth, plant phenotyping, disease diagnosis, weed infestation, insect damage, and food production, as well as emerging contamination with these agents. These data are then correlated to the site-targeted delivery, uptake, detection, retention, performance, interaction, and transformation of nanomaterial-based agrochemicals in plants.

### 1.3 Artificial Intelligence-Excelled SM Sensors

AI PA technologies have excelled in using self-powered wireless sensor networks (WSNs), weed patches, and PW sensors for remote and in situ monitoring.^3,23^ They involve nanosensors and nanomaterial-based delivery systems, PWs, and nanorobots that leverage online databases and algorithms of cheminformatics and translational bioinformatics.^3,18^ As a result, they enable spatiotemporal management of crops and livestock, low-cost in situ nutrient-sensing, precise agrochemical/fertilizer placement, and soil and plant tissue testing. By constantly measuring vegetative indices and evapotranspiration rates, AI PA helps control various and diverse plant growth conditions, nitrogen uptake, and secretion of nitrogen into the rhizosphere.

Before applying these sensors, one must overcome potential environmental pollution and mechanical breakdown risks. The plant-implantable or PW sensors should be constructed from biocompatible, mechanically resistant, nanotechnology-excelled materials, thus allowing well-defined interaction with microbiomes and metabolites exudated by plant leaves and roots.^33−35^ Although these sensors can be biotransformed or biodegraded in the phyllosphere, they are vulnerable to mechanical destruction, because of sudden weather changes. It is crucial to understand the (sensor material)-plant interactions, off-target performance, and long-term toxicity and persistence of the nanomaterial in the plant vascular structure and organelles, especially since the nanomaterials may penetrate the phloem and xylem, thus changing the fluid composition and flow rate.^18^ Finally, because of large-scale demands, energy storage costs must be well-considered. The efficient use of smart sensors requires efficient handling of enormous amounts of digitized output images and biophysical and physiological comprehension to solve real problems of scheduling sowing and controlling pesticide usage, as well as tracking and predicting crop growth and quality.^36,37^

Most advanced AI PA technologies propose intelligent self-powered WSNs and PW.^3,23^ Characterized by low-power demand, long-duration processing, and near-field communication (NFC), WSNs are coarsely distributed and interconnected sensors, typically exploited for environmental in situ and real-time monitoring. The WSNs, equipped with fully rechargeable batteries or battery-free solar energy harvesting systems for power supply, are easily controlled and power-supplied by smartphones, provided the distance from the source is less than several meters. However, they have limitations, including reduced light in the shaded, bushy, and foliaged places or prolonged signal transmission resulting from far distances from the aboveground microstrip antenna to the underground sensor.^38,39^ A similar technology underlies the modus operandi of PWs, i.e., the electronic devices implanted into the plant tissue for tracking and transmitting physiological parameters. Being an example of nanobionics, the PWs enable precise and continuous in-site or remote sensing of microclimate and tissue microenvironment changes, thus informing about the plant health and agrochemical performance.^40^

An outstanding recent review presents the state of the art of the nano biotechnologically excelled PW sensors.^23^ The present review focuses on developing analytical methods using conventional and SM-based sensors for GLP determination in water, food, crop, and soil samples. Expectedly, conventional sensors will soon be replaced by SM-based sensors. Examples of these sensors, including smartphone-integrated sensors, are briefly introduced and discussed herein.

## 2 Environmental and Human Toxicity of Glyphosate

### 2.1 Glyphosate Structure and Properties

Glyphosate, *N*-(phosphonomethyl) glycine (GLP), is a widely used broad-spectrum, nonselective, and postemergent herbicide for crop desiccation.^41^ Since the approval for agricultural use by U.S. Environmental Protection Agency (EPA) in 1974 and the authorization in the EU in 2002, GLP-based surfactants (GLP-SH) and GBHs have been commercialized as Roundup and RangerPro in over 140 countries, covering 350 million hectares of crops, with an annual turnover of 11 billion USD worldwide.^42,43^ According to the FAO, GBHs’ global market constitutes 18% of the total pesticide active ingredients (ActIs) and 92% of herbicide ActIs, with a global annual revenue accounting for 11 billion USD.^43^ As a powerful tool of modern crop management, prior-harvesting large-scale GBH interventions, called “green burndowns,” are expected to meet the growing demands for food and crops production, which are projected to increase to 100–110% by 2050.^41,44^

### 2.2 The Weed-Killing Activity of Glyphosate

GLP reversibly inhibits the 5-enolpyruvynyl-shikimate-3-phosphate synthase (EPSPS, EC 2.5.1.19) involved in the biosynthesis of aromatic amino acids in plants^45^ and some microorganisms.^46−49^ Roundup Ready crops are GLP-tolerant because they are genetically modified to carry the CP4 EPSPS gene derived from *Agrobacterium* sp. strain CP4, a naturally GLP-resistant rhizosphere bacteria.^46^ Moreover, the harmful efficacy of GLP against plant development strongly depends on the daily and circadian rhythms of the plant cells,^42^ and plants die within 4–20 days after crop spraying.^50^ This chronotherapeutic responsiveness of plants to GLP brings hope for optimized crop protection and safe food production security.

GBHs are commonly used in agriculture, industry, forestry, and weed management. From 1974 to 2014, the GBHs’ use increased 100-fold. However, the current regulations for the safety standards of GBH handling still rely on the studies performed in the late 1980s. Although the ∼90% growth of all GE seeds produced by Monsanto in 1996 is considered safe,^51^ and despite extensive research conducted in human biomonitoring, hazard assessments, epidemiological studies including occupationally exposed workers, pregnant women, and their offspring, and evaluation and standardization, the GBH safety to humans and the environment is still questioned.^52^

The major issues concern the environmental pollution that affects crops, soil, surface and groundwaters, sediment, and the spreading through wind and erosion, thus threatening wildlife and human occupational activities on farmlands and GBH factories.^53−56^ Various strategies for mitigating the global persistence and hazardous exposure to have been developed, as reviewed elsewhere.^54^

### 2.3 Human Toxicity of Glyphosate

The EPA-established daily chronic reference dose of GLP is 1.7 mg/kg of body weight, with a nonobservable adverse effect level of 50 mg/kg per day dose.^43,57^ Average urinary GLP levels for occupational exposure and nonoccupational exposure range from 0.26 to 73.5 μg/L and 0.13–7.6 μg/L, respectively.^58^ As an organophosphate (OP), GLP efficiently transmits orally, dermally, conjunctively, gastrointestinally, and via respiratory routes.^56^ According to the EFSA, GLP has low acute toxicity owing to the absence of the EPSP-metabolic pathway in vertebrates and the rapid degradation of GLP in mammals (half-life time of ∼5–10 h).^57,58^ However, occupational poisonings have become a global medical issue because of environmental pollution.^59^

Regarding genetic modification of GLP-tolerant crops, it is crucial to delineate the potential genotoxicity of the transgenic plants^60−63^ from the chemical toxicity of commercial GBH coformulants, including GLP isopropylamine (GLP-IPA) salt, polyoxyethyleneamine (POEA), and ppb traces of heavy metals, including chromium, cobalt, lead, or nickel^64,65^ and GLP metabolites, e.g., (aminomethyl) phosphonic acid (AMPA) and glufosinate.^65^ Because of the exquisitely disturbing impact of GBH surfactants on plant cell membranes,^66^ these are regarded as the most harmful GBH ingredients, demonstrated as more toxic than GLP alone in mammal cells.^67−69^

For example, a study on aquatic microorganisms (bacteria, microalgae, protozoa, and crustaceans) revealed the highest toxicity for POEA and GBH, whereas the only effect of GLP resulted from its acidity.^70^ Analogically, commercial GLP-SHs occurred more toxic than GLP-IPA in short-term acute toxicity trials (24 and 48 h).^71^ Moreover, in rats, 12-week exposure to GBH caused significant increases in kidney biomarkers, oxidative stress markers, and membrane-bound enzymes, indicating the accumulation of GLP residues in the kidneys, while GLP alone caused no nephrotoxicity.^72^

Concerning human toxicity, ingesting substantial GLP-SH volumes (100–500 mL) is associated with a human death rate up to 29.3%, depending on patients’ characteristics, such as age and intent of exposure.^73,74^ The uncoupling oxidative phosphorylation and POEA- or (heavy metals)-induced cardiovascular and cardiopulmonary harms are major lethal causes.^74−76^ According to a survey of medical reports of 107 patients, the ingestion of GLP-SH caused hypotension (47%), deterioration (38.6%), respiratory failure (30%), acute kidney injury (17.1%), and arrhythmia (10%). Interestingly, these complications depend on the volume of GLP-SH ingested and not the type of surfactant ingredient of the GBH.^77^ Emergency treatment of those intoxications comprises gastric lavage followed by hemodiafiltration and direct hemoperfusion, enabling the removal of GLP-IPA (228 Da) and surfactants (over 500 Da), respectively.^78^ Intensive care is required in severe GLP-SH intoxication, including dehydration, oliguria, paralytic ileus, hypovolemic shock, cardiogenic shock, pulmonary edema, hyperkalemia, and metabolic acidosis.^79,78^

## 3 Sensors and Separation Systems for GLP Determination

Nowadays, SM sensors are robustly applied to solve the most challenging environmental, socio-economic, and biomedical issues. Those involve rapid response to climate changes, real-time water, food, soil, and air quality monitoring, biotic and abiotic stress factors, nutrient recycling, sustained crop growth, biofuel production, point-of-care (POC) devices fabrication, and defense against bioterrorism.^22,80^ Receptor items of the modern SM sensors are emerging-field-deployable chemo- and biosensors and synthetic biology tools capable of detecting pathogenic pollution in various ecosystems and industrial environments. They involve molecularly imprinted polymers (MIPs), aptamers, viruses, prokaryotic or eukaryotic cells, and individual plants or insects that are able to sense single molecules or cells of emerging contaminants at pico- and femtomolar concentrations. Single-molecule/cell-based nanosensors allow determining agrochemical toxins as well as soil, plant, and insect-associated microbial communities. As such, they have become a powerful PA tool to track the impact of herbicides, pesticides, and insecticides on crop-associated microbiomes and ecosystems.^81,82^

Restricted concentrations of GLP in drinking water and food are 0.7 mg/L and 0.1–310 ppm, respectively.^83^ Various nanomaterial-based sensors for GLP have recently been prepared for detecting, adsorbing, and degrading GLP in real samples (Table 1). Conducting, semiconducting, and nonconducting polymers were prepared.^84^ For example, polyaniline-zeolite (PANI/ZSM-5 and PANI-FeZSM-5) composite-based adsorbents of efficient GLP adsorption capacity were prepared by oxidative polymerization.^85,86^ Polydopamine (PDA) was used to synthesize BiVO_4_/PDA-*g*-C_3_N_4_ photocatalyst sheets for exploiting dopamine self-polymerization, ultrasonic dispersion, and self-assembling. Under visible light irradiation, the BiVO_4_/PDAg-C_3_N_4_ photocatalysts degraded GLP more actively than the control composites prepared without PD.^87^ Fluorescent porous *N*-benzyl (carbazole derivative)-based polymer GLP detectors were synthesized in a one-step polymerization. The polymers of tunable pore sizes emitted bright cyan, blue, and green light upon ultraviolet (UV)-light excitation. GLP and other pesticides quenched the fluorescence of polymers according to the Stern–Volmer kinetics, thus demonstrating pesticide-specific recognition and determination.^88^ An intriguing report on a proteinoid polymer composite-based sensor for GLP was presented.^89^ The proteinoid polymer nanoparticles (NPs) were prepared by thermal step-growth polymerization of natural and unnatural amino acids in the presence of various agrochemicals. The agrochemicals interacted with the hollow NPs by encapsulation, integrating with the crude shell, or bound covalently/physically to the NP surface. Once hydrophobized and fluorescently labeled, the NPs were taken up by the plant and accumulated in the plant’s vascular system. This example shows the agrochemical-specific applications of these NPs to agriculture.^89^ Moreover, MIP-based GLP sensors of different properties and sensing parameters have been prepared.^90−103^ The current Review discusses several examples of these sensors (Table 2).

**Table 1:** Analytical Techniques for GLP Determination

analytical technique	LOD	LDCR	ref
FLD–HPLC	0.04 mg/kg	0.13–1000 mg/kg	(104)
ESI–MS–HPLC	0.01 mg/kg	0.04–1000 mg/kg	(104)
FLD–HPLC	0.01 mg/kg	0.005–0.5 mg/L	(109)
FLD–HPLC	0.6 μg/L	2–160 μg/L	(110)
LC–IRMS coupled with isotope labeling	<1 μM	n/a	(107)
HPLC with fluorescent labeling	0.02 ng/mL	1–3000 ng/mL	(111)
	0.002 mg/kg	n/a	
HPLC–MS	13 ng/mL	13–500 ng/mL	(105)
HPLC–ICP–MS/MS	8.2 μg/L	27–218 μg/L	(108)
HPLC-DAD	300 μg/L	1–8 mg/L	(108)
UPLC–MS/MS	0.05 μg/L	0.1–200 μg/L	(112)
MIP-based adsorptive-extracting systems	3.37 mg/mL	n/a	(90)
	n/a	BF: 2.12–2.33	(92)
	0.05 μg/L	Recovery: 96%	(97)
	0.043 μg/L	Recovery: 90.6–97.3%	(93)
	700 μM	n/a	(101)
SERS	<0.1 ppb	0.01–0.2 ppm	(118)
SERS sensor based on GO/AgNPs/Ti NT arrays nanocomposite	3 μg/L	0.005–50 mg/L	(120)
EPSP-based interferometric sensing	100 pM	10^–11^–10^–8^ M	(117)
Colorimetry	0.847 μM	1–40 μM	(121)
	1.23 μM	1–40 μM	
MIP-based luminescent determination	2 μg/mL	1–40 μg/mL	(103)
Porous-carbazole fluorescent determination	0.35 μM	n/a	(88)
DNA-labeled fluorescent magnetic core–shell NPs	0.27 nM	1–10000 nM	(116)
DNA-templated AuNC-based fluorescent determination	5 μg/L	15–100 μg/L	(122)
FRET fluorescent and colorimetric determination using SiNPs	0.003 μg/mL	0.15–1.5 μg/mL	(123)
Fluorescence of 4-butyl-3-thiosemicarbazide-labeled CDs	0.27 μM	0.4–30 μM	(124)
Fluorescence of 1,4-dihydroxyanthraquinone-labeled CDs	0.8 ng/mL	50–1300 ng/mL	(125)
Fluorescence of rhodamine B-embedded MOFs	0.18 μM	0.6–45 μM	(126)
Fluorescence of an UiO-67/Ce-MOF nanocomposite	0.0062 μg/mL	0.02–30 μg/mL	
Colorimetric/fluorescent/photothermal sensing by CDs-anchoring ferrocene MOF nanosheets	0.0131 μg/mL	0.039–3.19 μg/mL	(128)
	0.0015 μg/mL	0.0088–3.98 μg/mL	
Fluorescence-assisted immune-magnetic system	88.8 ng/L	0.0001–10 mg/L	(115)
FRET-assisted determination systems	9.8 ng/kg	0.02–2 μg/kg	(114)
	0.6 μM	0.02–2 μM	(119)
	0.79 μM	0.5–20 μM	(113)
Probe-free SPCE-mediated electrochemical sensing	2 μM	0.01–0.3 mM	(131)
Electrochemiluminescence	0.1 nM	0.1 nM–10 mM	(133)
HRP-based electrochemical sensor	1.7 μg/L	0.25–14 μg/L	(132)
Electrochemical sensing by enzymatic laser-induced graphene	3.03 μM	10–260 μM	(136)
(SWCNT/polyfluorene-bipyridine)-based water-gated transistor	1 nM	10^–2^–10^2^ μM	(137)
Amperometric determination using copper NP-based sensor	3.42 μM	0–25 μM	(138)
EIS/immunoassay	0.1 ng/mL	0.1–72 ng/mL	(141)
EIS/immunoassay-based two-plex sensing platform	1 ng/mL	0.3–243 ng/mL	(142)
HRP-modified-pencil graphite electrode for CV and amperometry	0.025 mg/L	0.1–10 mg/L	(140)
Electrochemical sensing based on Ti_3_C_2_T_*x*_/Cu nanocomposite	24 fM	0.1 pM – 1 μM	(139)
Machine learning-assisted electro-immunosensor	0.01 ppm	0.01–5 ppm	(130)
Urease-conjugated electrochemical sensing	0.5 ppm	0.5–50 ppm	(134)
Electroimmunochemical assay	5 ng/L	0–10000 ng/L	(129)
Electrochemiluminescent enzymatic immunoassay	0.032 mM	0.1–100 mM	(135)
MIP-based electrochemical systems	0.8 pg/L	1 pg/L −1 μg/L	(91)
	1 pM	1 pM – 1 μM	(95)
	0.27 ng/mL	5–800 ng/mL	(102)
	0.1 nM	0.1–100 nM	(94)
	0.35 ng/mL	3.98–176.23 ng/mL	(96)
	n/a	n/a	(143)
	4 nM	0.025–500 μM	(144)
	92 ng/L	400–1200 ng/L	(100)
AFM-assisted enzyme-based cantilever nanobiosensor	0.028 mg/L	0.01–10 mg/L	(145)
ELISA	0.05 μg/L	0.05–4 μg/L	(147)
	23 μg/kg	50–4000 ng/L (50–4000 ng/g)	(150)
GLP-ovalbumin conjugate-based ELISA	2 ng/mL	2–1000 ng/mL	(149)
HPLC–MS-assisted ELISA with online SPE	3.2 ng/L	50–500 ng/L	(148)
AuNP-oligonucleotide immuno-PCR sensing	4.5 pg/g	61.1 pg/g–31.3 ng/g	(146)
Fluorescent electrophoresis-coupled LOC	0.17 μg/L	0.17–845 μg/L	(152)
3D-μPAD-QD-MIP colorimetric-catalytic sensor	0.05–0.09 μg/L	0.5–50 μg/L	(151)
MIP-based microfluidic-electrochemical systems	0.002 μg/mL	0.005–50 μg/mL	(98)
	247 nM	0–1 mM	(99)
Smartphone-integrated lab-in-a-syringe sensor	2.81 nM	0–10 μM	(154)
Smartphone-integrated enzyme-free fluorimetric paper sensor	4.19 nM	0–180 nM	(156)
Smartphone-assisted colorimetric and fluorescent sensor	2.66 μM	0–30 μM	(155)
Smartphone-assisted fluorescence/colorimetric/SERS assay	0.738 nM	0–0.12 μM	(158)
	2.26 nM	0–220 nM	
	0.186 nM	0.5–12 nM	
In-field robotics-assisted in-row weed control	n/a	n/a	(159)
Smartphone-assisted AChE-based colorimetric sensor	0.15 μM	1.5 nM – 15 μM	(157)

**Table 2:** Analytical Parameters of MIP-Based Chemosensors for GLP Determination

functional monomer	cross-linking monomer	polymerization method	signal transduction technique	properties	ref
Acrylamide	EGDMA	T-FRP	Optical	LDCR: 5–40 μg/mL	(103)
				LOD: 0.046 μg/mL	
*N*-Isopropylacrylamide	BAA	T-FRP	Optical	LDCR: 0.005–50 μg/mL	(98)
				LOD: 0.002 μg/mL	
3-(4-Sulfonylbutyl)-1-[3-(triethoxysilyl) propyl]-1H-imidazolium	TEOS	Sol–gel polycondensation	Optical	weLDCR: 0.1 nM - 800 μM	(94)
				LOD: 0.1 nM	
*N*-Methacryl-l-cysteine	EGDMA	FRP, electropolymerization	Electrochemical	LDCR: 3.98–176.24 ng/mL	(96)
				LOD: 0.35 ng/mL	
Pyrrole		Electropolymerization	Electrochemical	LDCR: 1 pM – 10 μM	(95)
				LOD: 1 pM	
*p*-Aminothiophenol		Electropolymerization	Electrochemical	LDCR: 1 pg/L–1 μg/L	(91)
				LOD 0.8 pg/L	
Pyrrole		Electropolymerization	Electrochemical	LDCR: 5–800 ng/L	(102)
				LOD: 0.27 ng/mL	
Pyrrole		Electropolymerization	Electrochemical	LDCR: 400–1200 ng/L	(100)
				LOD: 92 ng/mL	
Pyrrole		Electropolymerization	Electrochemical	LDCR: n/a	(143)
				LOD: n/a	
Acrylic acid and *N*-vinyl-2-pyrrolidone	DHEBA	Electropolymerization	Electrochemical	LDCR: 0.025–500 μM	(144)
				LOD: 4 nM	

Various methods have been used to determine or extract traces of GLP and AMPA from liquids, solids, and plants (Table 1). Multimethod approaches, including chromatography,^104−112^ adsorption-extraction systems,^90,92,93,97,101^ optical,^88,103,113−128^ electrochemical,^54,91,94−96,100,102,129−144^ and scanning-probe techniques,^145^ immunochemistry,^146−150^ and microfluidic LOCs^98,99,151−153^ exploit modern GLP-sensitive materials to detect and analyze, as well as to extract and degrade, GLP traces. Hybrid nanomaterials used in these approaches include enzymes (AChE, ESPS), deoxyribonucleic acid (DNA)- or antibody-based aptamers, immune-magnetic conjugates, MIPs, and inorganic materials. Moreover, we herein discuss recent reports concerning the GLP determination by AI-excelled smartphone-integrated sensors,^154−158^ and an automated vegetable analyzer for targeted GLP delivery.^159^

### 3.1 Chromatography and Adsorptive-Extracting Systems for GLP

Chromatographic techniques are traditional tools for determining pesticides in liquids, foods, and clinical and environmental samples. They enable the pretreatment, separation, detection, or degradation of pesticides among other contaminants (micropollutants) of emerging concern, including endocrine-disrupting chemicals, plasticizers, artificial sweeteners, pharmaceuticals, personal care products, pyrethroid insecticides, and halogenated or organophosphorus retardants.^160−162^

For example, an intelligent postacquisition sample validation following mixed-mode solid-phase extraction (SPE) and ultraperformance liquid chromatography quadrupole-time-of-flight mass spectrometry (UPLC-Q-ToF-HRMS/MS) was employed for wide-scope target screening of 2316 emerging pollutants in wastewater samples collected from the Wastewater Treatment Plant of Athens. Upon validation of the method, it was employed to detect and quantify the influent and effluent wastewater connect of 398 selected contaminants of pesticides, opiates, and opioids, stimulants and sympathotomimetics, cannabinoids barbiturates, benzodiazepins, tranquilizers, analgesics, antibiotics, steroids, and industrial chemicals. This method allowed for determining the contents as low as 0.3 ng/L (perfluoroundecanoic acid), 0.4 ng/L (acetochlor, *N*-2,4-dimethylphenylformamide), and 0.5 ng/L (haloperidol, perfluoroheptanesulfonic acid).^163^ Moreover, HRMS-based suspect screening integrated with national monitoring data was recently applied in aquatic toxicology by investigating the presence of 16 not-well-explored pesticides and 242 pesticide transformation products in Swedish agricultural areas and streams. The study confirmed the occurrence of 11 transformation products and 12 tentatively identified ones.^164^

Furthermore, gas chromatography coupled to electron ionization mass spectrometry (GC-EIMS) was employed to determine levels of OP ethers in air and soil samples.^165^ Chromatographic analysis of hazardous agrochemicals usually involves two steps. First, the analyte is pretreated with optically active or polystyrene-coated magnetic NPs and then separated using GC or high-performance liquid chromatography (HPLC) coupled with a UV spectroscopic, fluorescent (FLD), or mass spectrometric (MS) detector. Most advanced adsorptive/separating chromatographic systems include nanotechnologically excellent adsorptive systems, engaging MIPs or silica NPs (SiNPs) of various porosity, developed surface area, and sorbing properties. Moreover, they are often modified with ionic liquids, silanes, amines, enzymes (AChE, carboxylesterases, laccases, or OP hydrolases), fluorescent, electrochemiluminescent, or surface-enhanced Raman spectroscopy (SERS) labels and magnetic beads. Finally, these systems are prepared as beads, wires, and sheets to improve their sensitivity, stability, amenability to modifications, and “on-site” applicability.^166,167^

Novel chromatographic techniques applied to GLP determination involve GC or HPLC coupled with UV spectroscopic,^106^ FLD, or electrospray ionization mass spectrometric (ESI-MS) detectors,^104^ often using fluorescent^111^ or carbon isotope (δ ^13^C) label probes.^107^ The HPLC-FLD and -(ESI-MS) determined GLP fluorenylmethyloxycarbonyl (FMOC) derivatives in maize in the 0.1–0.4 mg/kg range with the 79–86% efficacy of extraction recovery and the limit of quantification (LOQ) of 0.4 ng/mL.^104^ Similar FMOC-based HPLC methods determined GLP in soil^109^ and seawater^110^ with 0.01 mg/kg and 0.6 μg/mL LOD, respectively. With a different method, an HPLC precolumn was derivatized with 3,6-dimethoxy-9-phenyl-9*H*-carbazole-1-sulfonyl chloride (DPPC-Cl) to determine GLP in soybean with the LOD of 0.02 ng/mL and the extraction recovery exceeding 95%.^111^ The LC coupled to isotope-ratio MS (LC–IRMS) enabled the GLP determination in 21 commercial herbicide samples, revealing δ ^13^C values between −24 ‰ and −34 ‰ in the submicrogram concentration range.^107^ With porous (graphitized carbon absorbent)-based chromatography coupled to a three-quadrupole MS detector, the 6 ng/mL LOD of GLP in aqueous solutions was attained.^105^ In a similar study, with an HPLC coupled with an inductively coupled plasma (ICP-MS) detector or a diode array detector (DAD), GLP was determined with the LOD of 8.2 and 300 μg/L, respectively.^108^ Chromatographic analysis was employed for the GLP determination in real-life water samples from 10 agricultural provinces of China during various meteorological conditions.^112^ Specifically, UPLC-MS-MS was employed to provide the risk assessment and spatioseasonal distribution of GLP, AMPA, and glufosinate in China’s groundwater and surface water samples, from 2017 to 2018. GLP was determined with the LOD of 0.05 μg/L in the linear dynamic concentration range (LDCR) of 0.1–200 μg/L. Combined with the model simulations for potential leaching to water bodies, these quantitative results allowed us to establish that the spray drift deposition, runoff, and erosion are the main drivers of the aquatic GLP exposure from crop neighborhood, especially in summer and autumn seasons.

Inevitable progress will be made to address well-known limitations of GLP-targeted separating systems. Modern separation techniques, including capillary electrophoresis, GC, or LC, provide relatively low sensitivity (nanomolar) compared with optical, electrochemical, or immunochemical techniques that offer the detection of subnanomolar concentrations. Additionally, the blocky construction of modern systems disallows them for in-field use. Expectedly, the selectivity of future systems will be improved using tools of nanoinformatics, providing models and structures of analyte-receptor complexes of higher affinities. Finally, coupling these systems, based on aptamers, ionic liquids, NPs, and MIPs, with MS or FLD detectors, followed by their integration into the mobile microfluidic devices, shall enhance both sensitivity and applicability.^168^

#### 3.1.1 MIP-Based Chromatography of GLP

**Figure 1:**
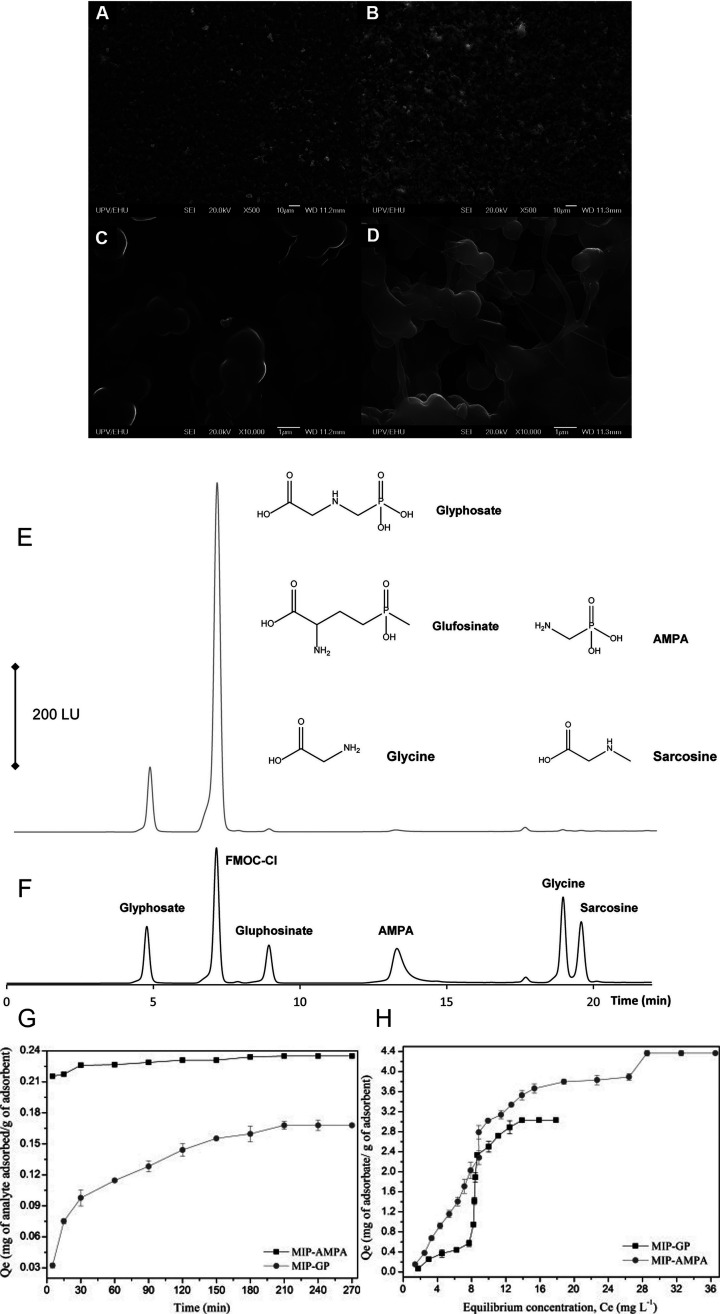
MIP-based adsorption systems for GLP separation and determination. (A-D) SEM micrographs of the surface of stir-bars coated with a DMAEM-ATU-EGDMA (A,B) NIP and (C,D) MIP-GLP. Chromatograms recorded after the injection of a solution of 100 μg/L GLP and all analogs (E) before and (F) after performing the MIP (stir-bar)-based extraction. Adapted with permission from ref (93). Copyright 2016 Elsevier. (G) Time-dependent adsorption of GLP and AMPA on the AA-EGDMA MIPs at pH = 6.5 and 25 °C. (H) Isotherms for MIP-GLP and MIP-AMPA at 25 °C. Adapted with permission from ref (90). Copyright 2014 Elsevier.

Various chromatographic methods developed to remove or degrade GLP traces from environmental, biological, or grocery samples exploited the adsorptive-extracting properties of porous MIPs.^169^ The maximum adsorption capacity of GLP-selective MIPs, formulated via free radical polymerization (FRP) of acrylamide (AA) and ethylene glycol dimethacrylate (EGDMA), was evaluated as high as 3.37 mg/g^90^ (Figure 1G and 1H). A series of dual-templated methacrylic acid MAA-MIPs, imprinted with herbicides, including GLP, were fabricated by precipitation polymerization for water treatment. Efficiency, expressed by binding factors (BFs), *K*_MIP_/*K*_NIP_, where *K* is the partition coefficient, of chosen GLP-selective MAA-MIPs in tap water for GLP solution, ranged between 2.12 and 2.55.^92^ Moreover, based on 1-allyl-2-thiourea (ATU), GLP-detecting MIP cartridges were prepared to assess the (UPLC-MS-MS)-mediated GLP recovery from mineral and underground waters. The herbicides were totally retained from these real matrices, spiked with 0.5 μg/L GLP.^97^ The selective sorptive extraction of GLP from river water and soil samples, with mean recoveries ranging from 90.6 to 97.3%, was demonstrated for ATU- and 2-dimethyl aminoethyl methacrylate (DMAEM)-based MIPs, prepared by UV light-activated FRP^93^ (Figure 1A-1F). Eventually, in a recent study, positively charged (quaternary ammonium cation)-MIP quartz crystal microbalance (QCM) sensors were constructed for the (electrostatic interaction)-mediated binding of GLP from river waters.^101^

The next-generation chromatographic sensors shall unite traditional multimodal separation and detection techniques, including thin-layer chromatography (TLC) or immunochromatography, with microfluidics and AI tools. Recent studies indicate the ultrasensitive on-site determination of toxins and pesticides in real samples using smartphone-coopted. Personal low-cost AI tools offer high-resolution photoimaging, portability, and instant online data availability that can be immediately shared. For example, an open-source smartphone-imaging app was developed to excel TLC screening and quantify pharmaceuticals.^170^ In another study, smartphone-based dual-channel immunochromatographic test strips labeled with polymer carbon QDs were fabricated for on-site simultaneous biomonitoring of cypermethrin, a pyrethroid pesticide, and its metabolite, 3-phenoxybenzoic acid, determined with LODs of 0.35 and 0.04 ng/mL, respectively.^171^ Expectedly, similar tools will soon be commercially available for GLP, as has already been demonstrated for other pesticides.

### 3.2 Optical Sensors for GLP

Recently, a series of optical strategies have been developed for pesticide sensing. Those include photonic, photoluminescent, photoelectric, electrochemiluminescent, and colorimetric methods. Over the past five years, carbon dot (CD)-based optical sensors equipped with aptamers, antibodies, enzymes, gold and silver nanoclusters (AuNCs and AgNCs), and nanoparticles (AuNPs and AgNPs), and MIPs as recognition units have been used as herbicide-derived signal indicators, catalysts, coreactants, and electrode surface modifiers.^169,172^ Moreover, novel carbon-based SERS biosensors with similarly excellent sensing properties were fabricated. They primarily include 0D carbon quantum dots (QDs), 1D carbon nanotubes (CNTs), 2D graphene, graphene oxide (GO), 3D carbon nanomaterials, and core–shell nanostructures. The SERS sensors were devised for selective and quantitative in situ analysis of agrochemicals by exploiting so-called localized “hotspots” produced during the application.^173^ QD-based chemosensors were used for the highly sensitive and selective detection of pesticide poisons in the clinical and forensic toxicological analysis of gaseous, anionic, phenolic, metallic, drug, and pesticide specimens. A breakthrough has been made by continuously applying whispering gallery modes (WGMs) to biosensing.^174−176^ Miniaturized size and excellent lasing properties of WGM-based microlasers, called resonators, were exploited in constructing label-free aptasensors^177^ and applied to detect single molecules, particles, cells, and molecular electrostatic changes at biointerfaces and barcode-type tagging and tracking.^178−180^ Furthermore, remarkable advances have been made in developing electrochemiluminescent (ECL) and photoelectrochemical sensors to analyze food quality. In most recent works, nanomaterial-based ECL luminophores have been synthesized and incorporated into immunoassay-, aptasensor-, and microfluidic systems for low-cost ultrasensitive determination of heavy metals, illegal additives, microbes, and pesticide contaminants in complex matrices.^181^ Food safety issues can be effectively solved by using brand-new photoelectrochemical biosensors. These devices, equipped with photoactive nanomaterial-based recognition units, quantitatively determine mycotoxins, antibiotics, and pesticides with high sensitivity at a significantly low signal-to-noise ratio.^182^

**Figure 2:**
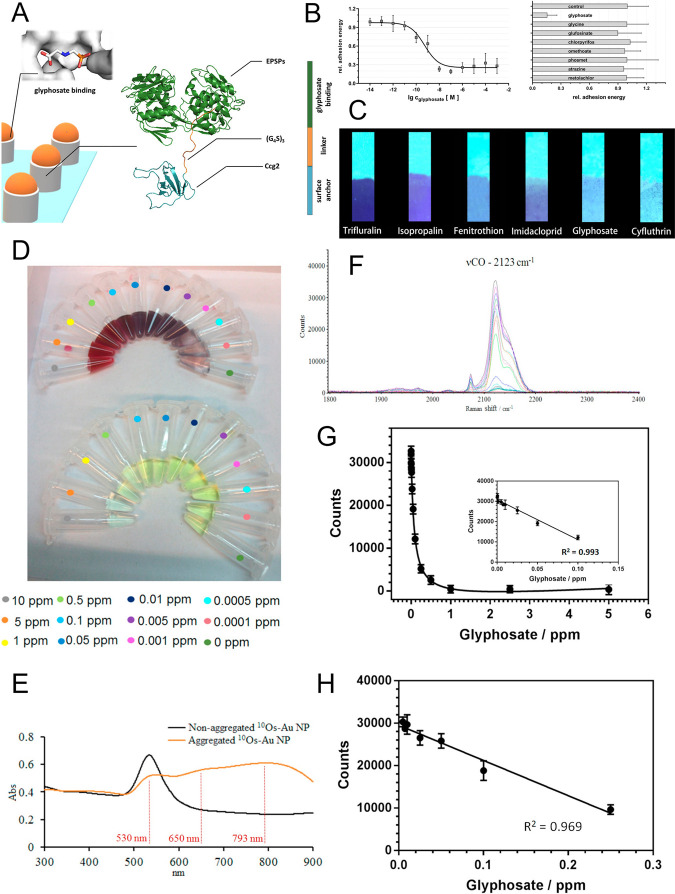
Optical sensors for GLP. (A) Scheme of a (glass slide)-adhered ESPS-based interferometric biochip (gray) specific for GLP (orange) deposited onto soft colloidal particles (SCPs), not shown. (B) Normalized relative adhesion energies of GLP-SCPs in the presence of GLP and other pesticides. (Left) GLP concentration dependence of adhesion energy. (Right) cross-reactivity of SCPs toward interferences. Adapted with permission from ref (117). Copyright 2020 Elsevier. (C) Strips of carbazole-based fluorescent porous polyaminals (PAN-C) test papers for sensing six pesticides in water at a concentration of 35 μM. Adapted with permission from ref (88). Copyright 2020 The American Chemical Society. A SERS sensor was used for GLP. (D) Dose-dependent colorimetric GLP determination by ^10Os^CO-AuNPs (top row), compared with a commercial standard (bottom row). (E) UV-NIR absorption spectra of nonaggregated and aggregated ^10Os^CO-AuNPs. (F) (GLP concentration)-dependent SERS spectra of ^10Os^CO-AuNPs (2123 cm^–1^). (G) GLP concentration dependence of the ν(CO) band intensity and (H) sensitivity assessment of the sensor in GLP-spiked beer samples. Adapted with permission from ref (118). Copyright 2020 Elsevier.

Modern optical methods of GLP sensing mainly exploit SERS, interferometry, chemiluminescence, colorimetry, fluorescence, and Förster resonance energy transfer (FRET). A SERS-based method, which exploits organometallic osmium carbonyl cluster-conjugated AuNPs, was used for AChE-mediated GLP determination with the LOD below 0.1 ppb^118^ (Figure 2D–2H).

Regarding SERS sensors for GLP, an air-stable sensor was devised by using reduced GO (rGO)-wrapped dual-layer AgNPs on TiO_2_ NT arrays as a SERS substrate. This sensor’s adsorption capacity and SERS enhancement were excellent thanks to the tremendous electromagnetic field and chemical enhancement generated by localized SPR excitation of the dense dual-layer AgNPs uniformly deposited onto the TiO_2_ NTs and to facilitation of the charge transfer between the extensive π–π conjugations in the rGO. Because the GLP molecule does not have a specific chemical group, it is hardly detectable using a conventional SERS method. In the discussed study, GLP was determined by the enhanced adsorption area of the nanocomposite. That enabled GLP determination in environmental samples of waters and soils in the LDCR of 0.005–50 mg/L with the LOD of 3 μg/L, i.e., lower than the limit specified by the U.S. EPA and the European Union.^120^

Two UV–vis colorimetric sensors for GLP were devised by linking 3-chloro-4-methylpyridine with 4-(dimethylamino) benzaldehyde or 4-(dimethylamino) cinnamaldehyde in a one-step synthesis, resulting in 4-(2-(3-chloropyridin-4-yl) vinyl)-*N*,*N*-dimethylaniline (BP-Cl) or 4-(3-chloropyridin-4-yl) buta-1,3-dien-1-yl)-*N*,*N*-dimethylaniline (CP-Cl), respectively. In the GLP reaction with these sensing compounds, the N atom of GLP interacted with the Cl atom on the pyridine ring, resulting in highly sensitive and selective naked-eye detection of GLP, observed as color changes ranging from colorless to yellow (BP-Cl) and from yellow to orange (CP-Cl). In a naked-eye analysis, GLP was determined in tap water and potato samples with the LOD of 15 and 10 μM for BP-Cl and CP-Cl, respectively, whereas using UV–vis spectrophotometry, these LODs were of 0.847 and 1.23 μM, respectively, in the LDCR of 1–40 μM.^121^

GLP was determined by using interferometry and chemiluminescence. Picomolar traces of GLP were detected using an EPSP-decorated interferometric sensor with GLP-attached poly(ethylene glycol) (PEG)-based soft colloidal probes^117^ (Figure 2A,B). In a chemiluminescent-assisted method of GLP determination, the LOD of 46 ng/mL was reached using poly(vinyl chloride) (PVC)-MIP microbeads prepared by FRP of acrylamide and subsequent conjugation with PVC.^103^

Fluorescent sensors rely on the analyte-induced triggering or quenching of the receptor’s fluorescence, called the “ON/OFF strategy.” In this strategy, pesticides, including GLP, act as either direct or indirect quenchers or triggers of fluorescence. In the direct approach, GLP binding by the receptor modifies its electronic structure and fluorescent properties, thus enabling direct optical sensing and determination of the GLP content. In contrast, in the indirect approach, GLP interacts with the molecular trigger/quencher to subsequently turn on/off the receptor. Recent studies on GLP-based fluorescence modulation demonstrate examples of both approaches.

GLP-induced fluorescence was directly quenched using carbazole-based porous polyaminals^88^ (Figure 2C). Moreover, various hybrid fluorescent-magnetic immunosensors were fabricated to detect GLP in liquids. For example, water-in-oil microemulsion-based Co–V/SiO_2_ NPs, doped with rhodamine, enabled immunoassaying GLP-dsDNA double target/probe core–shell NPs with the LOD of 0.35 nM.^116^ Likewise, a magnetic-assisted oligonucleotide aptamer probe labeled with 6-carboxy-fluorescein was used for GLP sensing with the LOD of 88.8 ng/L.^115^ Finally, recently devised FRET-based sensors for GLP explored GLP-induced turn-on fluorescence following the aggregation of positively charged cysteamine-AuNPs and negatively charged CdTe QDs capped with thioglycolic acid. This FRET assay utilized GLP detection in apples with a 9.8 ng/kg LOD.^114^ In another study, the fluorescent carbon QD probe operating in the AND logic gate was successfully quenched by GLP, resulting in the GLP determination with the LOD of 0.6 μM.^119^ Finally, the GLP-induced FRET switching in a self-assembled nanosensing system, formulated from *p*-*tert*-butylcalix [4] arene-grafted ruthenium(II) bipyridine-doped SiNPs, was used for GLP determination with the LOD of 0.791 μM.^113^

The indirect-modulation fluorescence sensor mainly relies on the chelating properties of GLP, provided by its phosphonate and carboxyl groups, as well as the monoprotonated secondary amine nitrogen atom. A Cu^2+^-modulated DNA-templated AgNCs was used to determine GLP by GLP reaction with the Cu^2+^, a primary quencher. Upon chelation, the DNA AgNCs fluorescence was recovered, which enabled the stoichiometric determination of GLP in real samples in the LDCR of 15–100 μg/L and with the LOD of 5 μg/L.^122^ A similar approach was exploited in fluorescent and colorimetric sensing of Cu^2+^ and GLP by the (*o*-phenylenediamine)-SiNPs FRET interaction. The Cu^2+^ oxidation of *o*-phenyaldiamine disabled the fluorescence of SiNPs by FRET. Hence, by chelating Cu^2+^, GLP served as a secondary quencher by hindering the FRET donor’s oxidation and restoring the FRET acceptor’s emission. This approach allowed determining GLP in the LDCR of 0.15 to 1.5 μg/L with the LOD of 0.003 μg/mL.^123^ Consistently, the concept of the Cu^2+^-GLP system was used in a fluorescent 4-butyl-3-thiosemicarbazide-labeled CDs-based sensor. Cu^2+^-quenched fluorescence of the sensor was recovered by the GLP addition in a dose-dependent manner, which enabled GLP determination in real samples with the LOD of 0.27 μM.^124^ Likewise, a Cu^2+^-modulated 4-dihydroxyanthraquinone-CD nanosensor enabled for ultrasensitive sensing of GLP in vegetable samples with the LDCR of 50 to 1300 ng/mL and the LOD of 0.8 ng/mL.^125^ Moreover, GLP was rapidly determined in real samples of agri-food products (tea, soybean, wheat, cucumber) using (rhodamine B)-embedded amino-functionalized iron-based MOFs bonded with Cu^2+^ via Lewis interactions that resulted in fluorescence quenching. The addition of GLP resulted in Cu^2+^ chelation via hydrogen bonding, thus turning on the fluorescence of the nanosensor. That allowed for the GLP determination with the LOD of 0.18 μM in the 0.6–45 μM LDCR.^126^ Similarly, a hierarchical, highly porous fluorescent nanocomposite, UiO-67/Ce-PC, consisting of UiO-67 NPs grown on Ce-MOF-derived porous carbon, was devised to determine GLP in soybean, wheat, and corn. A large specific surface area and abundant metal active sites that alleviated the diffusion barrier and enhanced the GLP preenrichment ensured the determination. Additionally, the competitive coordination effect between GLP’s phosphonate groups and metalloorganic ligands (hydroxyl groups) attenuated the ligand-to-metal charge transfer between metallic nodes and organic struts, thus providing a dose-dependent fluorescence recovery upon GLP detection. The plot of the fluorescence enhancement response of UiO-67/Ce-PC toward GLP was linear in the range of 0.02–30 μg/mL with the LOD of 0.0062 μg/mL.^127^

An optical/temperature nanozyme platform, fabricated from nitrogen-doped CDs anchored onto Zr-based ferrocene MOF nanosheets, was devised for the sensitive and portable determination of GLP. The nanozyme mimicked peroxidase activity, i.e., oxidation of colorless 3,3′,5,5′-tetramethylbenzidine into a blue product in the presence of H_2_O_2_, which GLP readily suppressed. That enabled a trisignal response of fluorescence enhancement, absorbance, and temperature decrease. These features were exploited to construct a portable mini-photothermal device capable of colorimetric and fluorescent GLP determination in the 0.039–3.19 μg/mL LDCR with the LOD of 0.0131 μg/mL and the 0.0088–3.98 μg/mL LDCR with the LOD of 0.0015 μg/mL, respectively.^128^

Because of the analyte-triggered AChE inhibition, the optical sensors for GLP, based on the single molecule “turn on/off” on-site sensing mode, belong to the most sensitive and selective. Future optical technologies, compatible with smartphone-assisted kits and LOCs, include paper-, liquid-, and gel-based sensors. Double-signal fluorescence, phosphorescence, chemiluminescence, lateral flow immunoassay, or enzymatic fiber-optic biosensing are envisioned as the most promising operation strategies, enabling the in-field red-green-blue (RGB)-determination of GLP. Most recent advancements in optical sensors involve metasurface-based devices. Metasurfaces are 2D composite micro- or nanomaterials of the subwavelength thickness and desired geometry, allowing for adjusting the material’s refractive index of the material to positive, near-zero, or negative values (so-called negative refraction). Moreover, the nonlinear metasurface can transform the infrared signal into the visible signal, which the human eye or smartphone CCD can visualize.^183^ These exquisite features allow for devising a surface-enhanced infrared absorption-operating sensor for passive trapping and detecting glucose and proline with the LOD of ∼1 pg.^184^ In this context, the LODs of the devices discussed in the present review are comparable or one order of magnitude lower. Besides, devising optical sensors for agricultural use shall necessitate integrating ultrasensitive (of the order of ∼fM) devices with smartphones or portable meters, similar to commercial smart Raman spectroscopic alcohol meters.

### 3.3 Electrochemical Sensors for GLP

The contemporary electrochemical sensors provide a portable, simple, and sensitive determination of herbicides and pesticides in real water, food, and soil samples.^185,186^ Recently fabricated multimodal sensing devices contain aptamers, enzymes, graphene, CNTs, polymers, viruses, and cells as recognition units integrated with optical, piezoelectric, and microgravimetric transducers.^187,188^ The GLP concentration is mainly measured using amperometry, cyclic voltammetry (CV), differential pulse voltammetry (DPV), nonfaradic electrochemical impedance spectroscopy (EIS), and electrochemical surface plasmon resonance (SPR) spectroscopy.

Among other OP pesticides, including glufosinate and AMPA, GLP was determined amperometrically using a three-electrode sensor fabricated by (UV laser)-inscribing of OP-selective Cu NPs on a polyimide film. For GLP determination in natural water samples, the sensor exhibited the LOD of 3.42 μM, whereas for glufosinate and AMPA, the LOD was 7.28 and 17.78 μM, respectively. Moreover, the sensor prevailed in pesticide selectivity in the presence of ion and organic interferences in natural water.^138^ Recently, a very sensitive, highly conducting sensor for GLP was constructed based on Cu-benzene-1,3,5-tricarboxylate-loaded 2D Ti_3_C_2_T_*x*_ nanosheets deposited on a glassy carbon electrode (GCE). This nanocomposite was fabricated upon in situ copper component growth on chemically etching nanosheets that were subsequently dispersed and vacuum-dried on the GCE. Because of the Cu ions’ high affinity to GLP, the sensor sensitivity was excellent, resulting in the exquisitely low LOD of 26 fM and a broad LDCR of 100 fM to 1 μM.^139^

Carbon-based electrochemical sensors have recently become a powerful tool for tracing metabolism. The significant advantage of these materials is the enhancement of the electrochemical sensing performance by enlarging an active surface area. For example, a probe-free screen-printed carbon electrode (SPCE) was used for direct GLP sensing in tap water with micromolar sensitivity.^131^ An ECL horseradish peroxidase (HRP)-based sensor, formulated on a sulfonate polymer matrix, was used for the GLP determination with the LOD of 1.7 μg/L.^132^ In a recent study, a simple and accurate (pencil graphite electrode)-supported sensor containing an HRP enzyme, immobilized on a multiwalled CNTs (MWCNTs)-doped polysulfone membrane, was devised to detect GLP in the river and drinking water samples. This highly selective sensor was readily applied to the in-field determination of GLP, showing reproducible and repeatable CV and amperometric readouts in the LDCR of 0.1–10 mg/L and the LOD of 0.025 mg/L.^140^ Moreover, functionalized single-walled CNT (SWCNT)-based nanomaterials were used in electrochemical GLP sensing by providing transducing layers for water-gated transistor-based sensors. In particular, GLP was selectively determined with a sensor composed of networks of semiconducting, monochiral (6,5) SWCNTs featured with polyfluorene-bipyridine copolymer and a Cu^2+^-selective membrane. The functionality of this semiconducting sensor relied on the n-doping resulting from Cu^2+^ complexation by bipyridine. Adding GLP suppressed this complexation by competitive chelation of Cu^2+^, thus enabling the stoichiometric quantification of the herbicide analyte at nanomolar concentrations.^137^

Likewise, GLP was selectively determined using an amperometric sensor containing glycine oxidase, a flavoenzyme, immobilized on a platinum-decorated laser-induced graphene scaffold. Exquisite electronic and solid properties, including the LDCR of 10–260 μM and the LOD of 3.03 μM, allowed for selective determination of GLP with only minimal interference of common herbicides and insecticides, including atrazine, 2,4-dichlorophenoxyacetic acid, dicamba, parathion-methyl, paraoxon-methyl, malathion, chlorpyrifos, thiamethoxam, clothianidin, and imidacloprid. In the future, the sensor may enable food mapping and determining GLP in complex river waters and crop residue fluids.^136^

GLP was sensed by using electrochemical immunoassays. Recently, a label-free, portable, selective, and highly sensitive (LOD of 0.1 ng/mL in the LDCR of 0.1–72 ng/mL) sensor was devised to determine GLP in human urine.^141^ Its electrochemical platform consisted of a portable printed-circuit circular board with gold working and reference electrodes enabling nonfaradic EIS measurements. Its immunoassay-based platform included a monolayer of dithiobis(succinimidyl propionate), a thiol-based cross-linking monomer modified with a GLP antibody, and a coated gold electrode. The selectivity was assessed using typical herbicide interferences, including malathion, 3-phenoxybenzoic acid, and chlorpyrifos.^141^ Likewise, GLP and chlorpyrifos were determined in low- and high-fat food matrices using a two-plex, portable electrochemical-immunoassay nonfaradic ESI-based sensor. Both sides of this sensor were functionalized with the respective antibody. In low fat, the sensor determined GLP and chlorpyrifos with a 1-ng/mL LOD in the 0.3–243 ng/mL GLP/chlorpyrifos LDCR, whereas in high fat, the LOD was 1 ng/mL in the LDCR of 1–243 ng/mL.^142^

**Figure 3:**
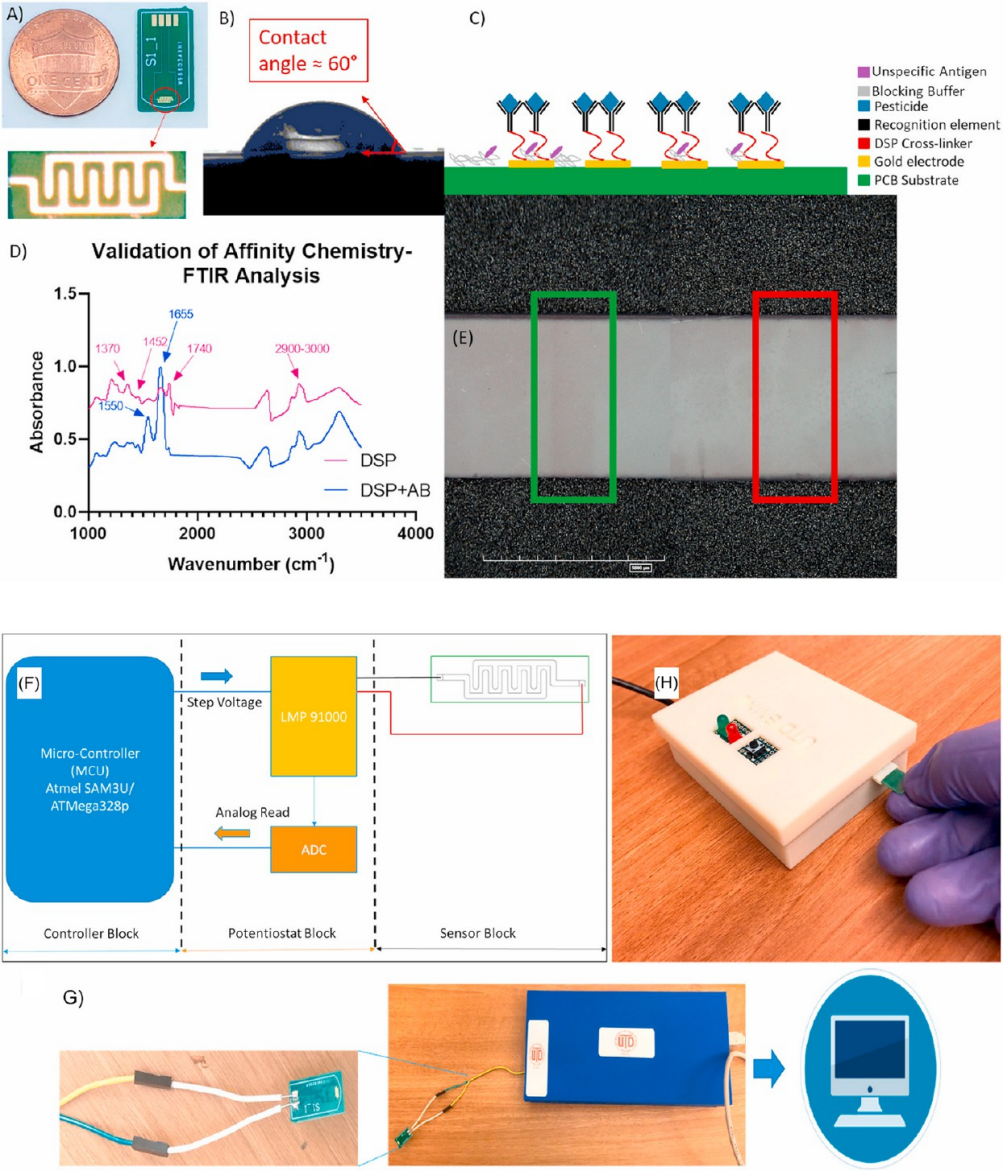
Field deployable ElectrochemSENSE Platform for GLP sensing. (A) An ElectrochemSENSE sensor strip. (B) Solid–liquid interface contact angle generated by phosphate-buffered saline on a sensor substrate. (C) Scheme and (D) FT-IR spectra representing the sensor electrochemical immunoassay chemistry. (E) Immunochromatographic test results displaying a faint test line (GLP-free, green) and the absence of a test line for a 100 ppb GLP sample (red). (F) A scheme depicting the functionality of the ElectrochemSENSE Platform. (G) Custom electronic platform variant to connect to a personal computer for data acquisition and processing. (H) Custom electronic platform variant for determining the produced SAFE/UNSAFE response. Adapted with permission from ref (130). Copyright 2020 Elsevier.

GLP was sensed with the LOD of 0.11 nM using sophisticated ECL sensors based on HRP-assisted in situ generations of ZnS QDs on ordered mesoporous carbon substrates.^133^ A bioconjugate of urease-AuNPs and an agarose-guar gum-entrapped biocomposite membrane was devised to detect the enzyme-inhibiting activity of GLP, enabling the determination of this herbicide in ppm traces.^134^ Similarly, GLP was determined with a sensitivity of 0.01 ppm (10 ng/mL) in fruits and vegetables using a field-deployable electrochemical immunosensor based on a polymer-metalized interdigitated two-electrode system, functionalized with a commercial GLP-antibody, and equipped with a portable reader and a machine-learning binary classifier^130^ (Figure 3). Likewise, an SPCE immunosensor, enabling the GLP determination with the 5 ng/L LOD, was fabricated using anti-GLP-IgG-modified magnetic beads and an HRP-conjugated-GLP tracer.^129^ Moreover, an electrodeposited monocrystalline silicon-PANI-HRP conjugate was constructed to evaluate an immunoassay for GLP sensing with the LOD of 5.44 μg/L.^135^ Continuously, a series of GLP-imprinted polymer-based electrochemical sensors was fabricated. Electropolymerized GLP-imprinted *p*-aminothiophenol metallorganic framework (MOF) films formed on AuNP surfaces determined GLP with a 0.8 pg/L LOQ.^91^ The LOD of 1 pM was determined using gravimetric-electrochemical GLP-selective polypyrrole (PPy) MIP films.^95^ Similar sensing efficacy (0.27 ng/mL) was demonstrated for an (Au-electrode)-deposited PPy-MIP sensor for GLP.^102^ Furthermore, in a recent study, a gold chip/electrode coated with a self-assembled monolayer of 11-(1*H*-pyrrol-1-yl) undecane-1-thiol, and PPy-MIPs, was exploited to determine GLP using CV, EIS, and electrochemical SPR analyses. Values of the dissociation constant, *K*_d_, and free Gibbs energy change, Δ*G*_0_, accompanying the interaction of GLP with MIP-PPy were determined as *K*_d_ = 38.18 (±2) × 10^–5^ and Δ*G*_0_ = −19.51 (±0.2) kJ/mol.^143^ In a similar study, a graphite SPCE modified with a dual-MIP coated on an amino-mesoporous SiNPs-PtNPs core was used for DPV determination of paraquat and GLP. The herbicide-selective MIPs were fabricated by using a 3D-surface imprinting strategy that increased the conductivity and monodispersity of the sensor. A dual-MIP was fabricated by 3D-printing on the surface of mesoporous SiPtNPs, using acrylic acid and *N*-vinyl-2-pyrrolidone as functional monomers, and *N*,*N*′-(1,2-dihydroxyethylene) bis(acrylamide) (DHEBA) as the cross-linking monomer. The sensors allowed for simultaneous determination of both herbicides, paraquat, and GLP, in water samples in the LDCR of 0.025–500 μM and with the LOD of 3.1 nM and 4 nM, respectively.^144^

A typical base-catalyzed sol–gel transition incorporation was performed to incorporate GLP and graphene QD labels into mesoporous organosilica MIPs. The GLP-induced quenching-mediated detection enabled GLP quantification at a subnanomolar level (0.017 ppb, 17 pg/mL).^94^ GLP was determined with the 0.35 ng/L LOD using a pencil graphite electrode dip-coated with AuNPs, then modified with the fabrication of glyphosate-glufosinate double-template MIP using atom transfer radical polymerization in the presence of templates and MWCNTs.^96^ Finally, GLP was ultrasensitively (the LOD of 92 ng/mL) determined by DPV using a composite of urchin-like AuNPs, Prussian Blue, and PPy-based GLP-MIPs, deposited by electropolymerization on the indium–tin oxide electrode.^100^

Electrochemical sensors for GLP are advantageous because of their excellent reproducibility and accuracy as well as high selectivity and sensitivity with a linear output among others. However, these features may deteriorate over time, as extended exposure to the target analyte usually shortens or limits the sensor lifetime, especially at variable temperatures. Once incorporated into the smart sensor, this device would require temperature compensation, which exploits the battery energy losses. Regarding sensitivity enhancement, biomimetic chemistry tools will measure the subfemtomolar GLP concentrations. Expectedly, the AChE-based nanozymes, containing MOFs and transient metal complexes, will be used to construct future sensors for GLP. In conclusion, although only a few examples of GLP-sensitive electrochemical smartphone-assisted sensors of subnanomolar LOD have so far been demonstrated, fabricating such tools is highly expected in the near future. For example, an (yttrium ferrite garnet)-embedded (graphitic carbon nitride)-based electrochemical POC sensor, integrated with a smartphone, was recently reported to determine pesticide mesotrione in food with the LOD of 0.95 nM.^189^

### 3.4 (Atomic Force Microscopy)-Based Sensors for GLP

Despite extreme usefulness, robustness, and sensitivity, atomic force microscopy (AFM) has hardly been used as a sensing tool. The main reasons included dimensions and the complicated curvature of the AFM tips. Recently, tip functionalization with various species has been optimized so that AFM has become an efficient technique for quantitatively determining chemicals, including herbicides and pesticides. Recently, atomic force spectroscopy, an AFM-derived technique, has been exploited to analyze imazaquin, metsulfuron-methyl, and atrazine samples using tips functionalized with the acetolactate synthase and antiatrazine antibody. This tip functionalization increased markedly (over 130, 140, and 175%, respectively) the adhesion force between the functionalized tips and the herbicides, distinguishing nonspecific and specific interactions between the tip-located biomolecules and the herbicides quantitatively.^190^

Regarding AFM-assisted GLP determination, a peroxidase-based AFM nanobiosensor was devised to evaluate GLP content in 0.01 to 10 mg/mL in zucchini extracts. This biosensor for GLP modus operandi relied on detecting GLP-induced changes in surface tension caused by GLP adsorption, followed by a conformational change in the peroxidase structure. The LOD was as low as 0.0238 mg/L.^145^

Low sensitivity, long scanning time, limited spatial resolution, and tip damage, i.e., significant drawbacks of AFM sensors, are expected to be addressed in the future. The construction of tuning-fork-balanced tips shall enable chiplike probing of biological and soft material samples. Applying tip-synchronized time-resolved electrostatic force microscopy will also allow for monitoring charge generation, transfer, and recombination, which is crucial for ultrasensitive enzymatic GLP determination in real samples.

### 3.5 Immunoassays and Immunosensors for GLP

**Figure 4:**
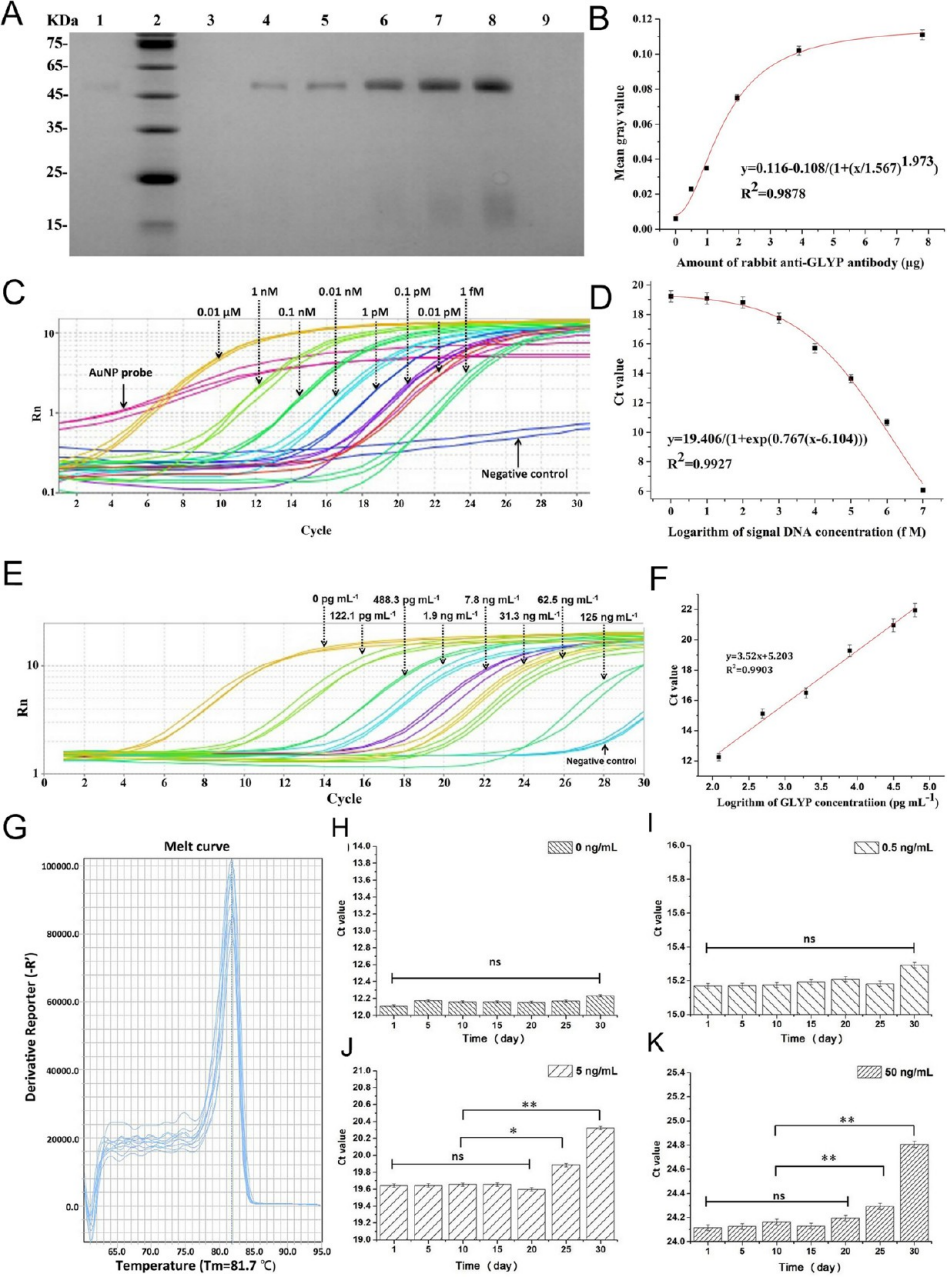
Oligonucleotide-functionalized AuNP probe-based biobarcode immuno-PCR sensor for GLP. Characterization of the AuNP probe by (A) (sodium dodecyl sulfate)-(polyacrylamide gel) electrophoresis, SDS-PAGE, quantification (lanes 4–8: antibody at 0.49, 0.98, 1.95, and 7.81 μg, respectively) and (B) immunoassay using anti-GLP antibodies. (C) Amplification curves of real-time PCR and (D) the standard curve acquired for DNA samples in the 1 fM to 0.01 μM concentration range. (E) Amplification curves of real-time PCR and (F) the standard curve for the sensor using aqueous GLP solutions. (G) Melt curves of real-time PCR and (H–K) sensor stability over 30 days upon determining at 0, 0.5, 5, and 50 ng/mL GLP. Adapted with permission from ref (146). Copyright 2021 Elsevier.

Immunosensors are (affinity-ligand)-based sensing tools exploiting immunochemical antibody–antigen interactions. These interactions are quantified by transducers with immobilized antibodies. Over decades, the use of immunosensors, both labeled and nonlabeled, has become highly trending, as they comprise (single molecule)-operating sensing tools for food safety control, healthcare, and environmental monitoring.^191^ Usually, forming an immunocomplex with an analyte generates electrochemical, photochemical, and piezoelectric changes, contributing to multimodal and sensitive detection.^192^ Herbicides and pesticides belong to typical immunosensor analytes, as they are easily complexed by peptides and enzymes immobilized on the transducer surface. The affinity-based reactions resemble physiological and biochemical reactions because these analytes act as natural ligands.^193^ Besides, using MIPs, well-known as “plastic antibodies,” “semisynthetic enzymes,” and “artificial receptors,”^194^ enables low-cost pesticide residue determination in foods, feeds, medicines, and environmental samples.^195^ Various immunosensors have been constructed for GLP sensing. These include antibody- and aptamer-based immuno-assisted electrochemical and optical sensors.^54^ Most recent examples report conventional immunochemical methods for GLP determination using enzyme-linked immunosorbent assay (ELISA), which provides submicromolar LODs in liquids and animal feeds.^147,150^ The ELISA combination with an online SPE, followed by HPLC–MS analysis, enabled the GLP determination with the subnanomolar LOD.^148^ Advanced immunoassays utilize an immobilized GLP-ovalbumin conjugate and avian IgY antibodies for sensing ppb traces of GLP^149^ or an AuNP oligonucleotide-based biobarcode immuno-(polymerase chain reaction) (PCR) system with the LOD of 4.5 pg/g^146^ (Figure 4).

Although contemporary immunosensors for GLP provide ultrasensitive determination, future trends promise the construction of sensors of higher standards. Because of the high production cost of contemporary biologically derived antibodies and enzymes, future immunosensors will be constructed from single atom/molecule or peptoid/enzyme mimetic catalysts containing single noble metal NCs or MOF nanozymes as recognition units. Since immunodetection of various environmentally emerging pesticide residuals can be executed with extremely low LODs (<pM),^196^ it is expected that the application of AI-excelled tools shall upgrade these values. As it was extensively summarized elsewhere,^197^ several smartphone-assisted immunosensors were so far devised for determining various biomedically or environmentally relevant analytes, including small molecules, macromolecules, viruses, and bacteria.

### 3.6 Microfluidic Lab-on-Chips for GLP

In recent decades, enormous progress has been made in devising microfluidic-assisted conventional and smart sensors. Technological progress enables microfabricating and miniaturizing microfluidic paper-based analytical devices (μPADs)^198^ and devising smartphone-coopted sensors operating in a microfluidic mode.^199^ In a traditional design, μPADs are constructed from conventional dipstick or lateral-flow setups. Once coupled with electrochemical immunosensors and LOCs, these eco-friendly devices have become onsite quantitative and semiqualitative equipment for POC medical diagnostics, food safety control, and environmental purity inspection. Particularly, microfluidic-assisted analytical devices enable direct, low-cost, sensitive, and real-time screening of chemical hazards or pathogens, including metal ions, nitrates and nitrites, phenols, pesticides and herbicides, and bacteria.^200−203^ Thanks to an impressive advance in biotechnological sciences, sophisticated living-cell-based microfluidic-handled biosensors have also been devised. They are mainly inspired by conventional in vitro bioassays, including the bacterial luminescence toxicity screen and the algal toxicity test by imaging pulse amplitude modulated fluorometry, which was demonstrated useful for pesticide determination in environmental samples.^204^ In the most advanced approach, algae-based biosensors sensitively, sustainably, and multiplexed analyzed agro-environmental samples. In these biosensors, whole algal cells and their photosynthetic complexes were used as miniaturized transducers to construct biomicrofluidic devices.^205^

**Figure 5:**
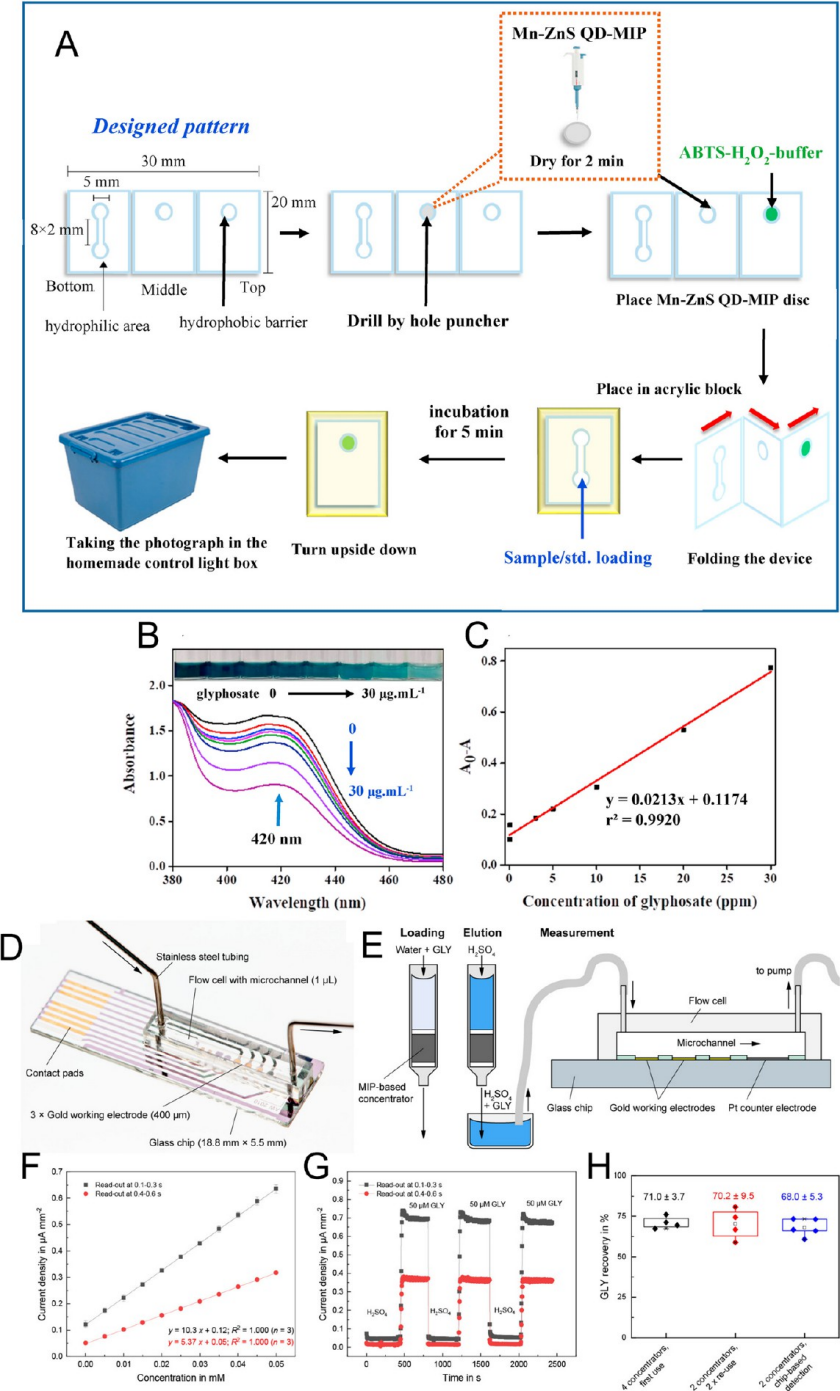
Multimodal microfluidic devices for GLP determination. (A) Colorimetric determination of GLP using Mn–ZnS QD-embedded MIPs combined with a 3D-μPAD, using a foldable sheet comprising three parts (top/center/bottom). (B) UV absorption spectra and (C) GLP calibration plot constructed based on the colorimetric determination of 0, 0.01, 0.05, 3, 5, 10, 20, 30 μg/mL GLP. Adapted with permission from ref (98). Copyright 2021 Elsevier. (D) The device and (E) cross-sectional view of the microfluidic MIP-based sensor with electrodeposited Au working electrodes and a poly(methyl methacrylate) flow cell with inlet and outlet channels. (F) Calibration curves and (G) continuous, reversible, and reproducible microfluidic chip-based determination of GLP. (H) A consistent (∼70%) electrochemical recovery of GLP from different MIP-based concentrators. Adapted with permission from ref (99). Copyright 2021 The American Chemical Society.

Modern microfluidic-handled LOC sensors combine different methods, e.g., electrophoresis-coupled disposable microchips with laser-induced fluorescence detection for the rapid on-site interference-free determination of GLP residues in agricultural products, with the LOD of 0.34 μg/L and 84% recovery.^152,153^ (Direct injection)-UPLC with triple quadrupole MS was optimized to determine GLP traces in environmental waters with the LOD range of 0.05–0.09 μg/L and 76.3% recovery.^151^ Moreover, Mn-ZnS QD-embedded MIP, combined with a 3D-μPAD sensor, was fabricated for colorimetric-catalytic determination of GLP. Selective recognition of GLP by the poly(*N*-isopropylacrylamide) (PNIPAM)- and *N*,*N*′-methylenebis(acrylamide) (MBA)-based MIPs, formulated by thermally induced FRP, enabled selective GLP determination in whole grain samples with 0.002 μg/mL LOD^98^ (Figure 5A-C). Finally, an electrochemical LOC-assisted commercial MIP-based sensor for GLP was devised. It enabled rapid, online, and real-time determination of GLP in tap water with the LOD of 247 nM^99^ (Figure 5D-H).

The next steps in devising future GLP-selective microfluidic-assisted sensors involve increasing sensitivity and integrating these sensors with AI tools including smartphones and mobile meters. These sensors will enable on-site ultrasensitive determination of GLP in foods, industrial waters, and biological fluids. Based on an ELISA assessment, such a tool has already been devised for on-site quantifying ppb (μg/kg) levels of aflatoxin B1 in moldy corn. The immunoassay sensor was 3D-printed on a plastic chip attachable to a smartphone.^206^ Similar sensitivity was achieved for various pesticides using a portable, smartphone-adaptable origami μPAD-based potentiostat. Relying on the pesticide-inhibited activity of the enzyme immobilized on the sensor’s transducing item allowed for chronoamperometric monitoring of paraoxon, 2,4-dichlorophenoxyacetic acid, and atrazine at a ppb level in river water samples.^207^

### 3.7 Smart Sensors and Plant Wearables for GLP

**Figure 6:**
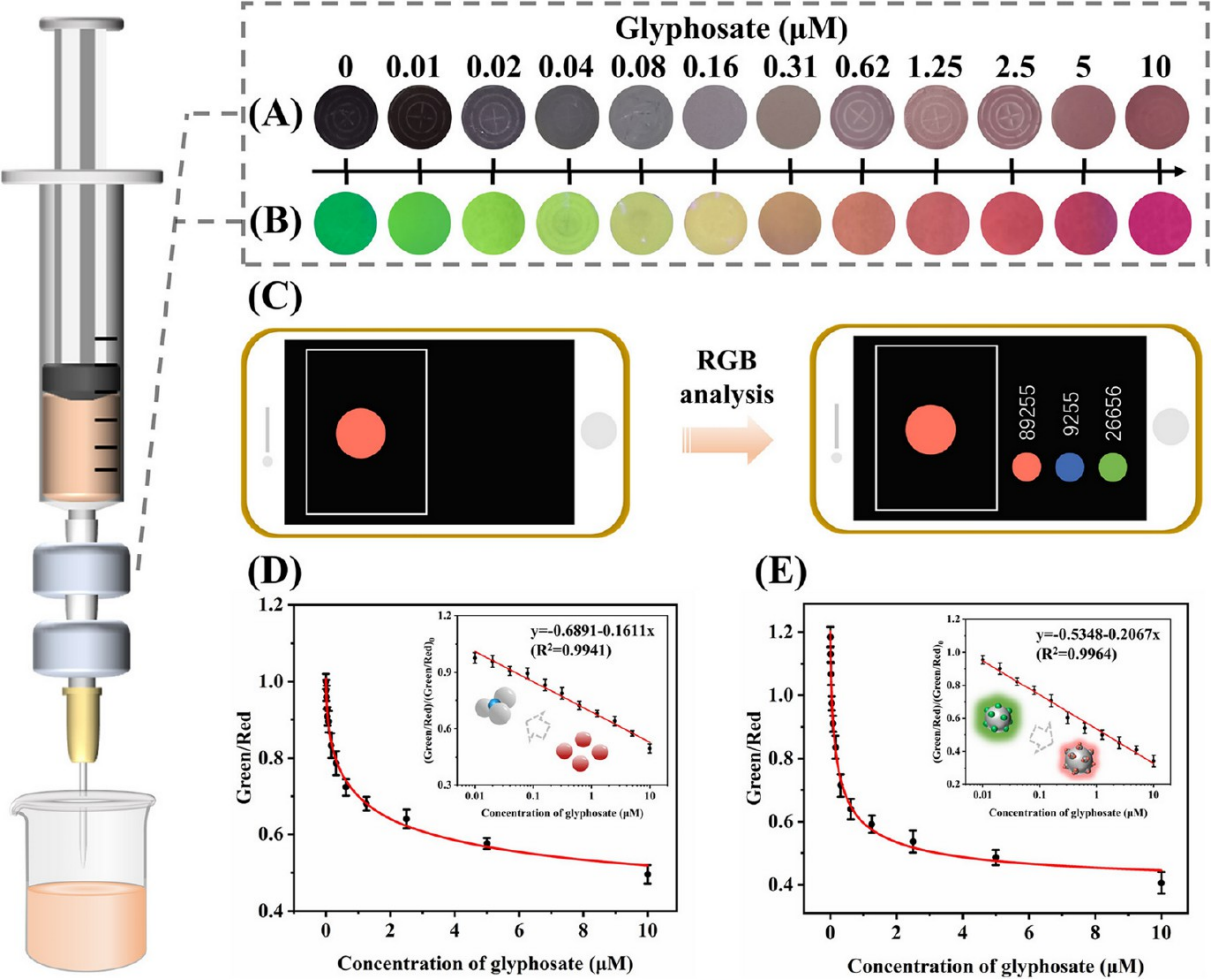
A smartphone-integrated lab-in-a-syringe device for the colorimetric and fluorescent on-site determination of GLP. (A) Colorimetric and (B) fluorescent imaging of rQDs-SiO_2_-gQDs+CTAB-AuNPs+AChE+ATch under daylight in 0 to 10 μM GLP. (C) RGB analysis of the fluorescence image using a smartphone color identifier application. (D) The ratio of green-to-red channel values of colorimetric and (E) fluorescent images in dependence on 0 to 10 μM GLP concentration. The respective insets depict plots of the ratios of green-to-red channel values versus 0–10 μM GLP concentration. Adapted with permission from ref (154). Copyright 2021 American Chemical Society.

The necessity of developing the PW pest-, insect-, fungi-, and herbicide-specific sensing and delivery systems is crucial because of the off-target toxicity of these biocides. Among heavy metals, antibiotics and other drugs, food-derived growth factors, and industrial hydrocarbon wastes, these biocides are the most toxic environmental pollutants and hazards to human health.^3^ Because of various leakage from the target zone to the rhizosphere, waters, and air, relatively high half-time biocides destabilize the trophic chain’s matter cycle and endanger ecosystem safety.^208−210^ Thus, enormous progress has been made in AI-enhanced mobile or PW biocide-selective sensors.^3,18^ Miniaturized smart sensors, incorporated, e.g., in smartphones, PWs, and POCs or field-deployable devices, are equipped with nanophotonic antennas and dielectric metasurfaces that enable few-molecule sensitivity by confining incident light into intense hotspots of the electromagnetic fields, thus delivering strongly enhanced light-matter interactions.^211^ Regarding GLP sensing, an in-smartphone-incorporated mobile lab-in-a-syringe platform was designed for the rapid, visual, quantitative determination of organophosphorus pesticides via dual-mode colorimetry and fluorescence measurements. The platform was based on (cetyltrimethylammonium bromide)-coated NPs conjugated with a silica pad modified with red- and green-emission QDs. In the sensing reaction, thiocholine, the product of AChE-mediated hydrolysis of thioacetylcholine, induced the aggregation of NPs, thus giving rise to the color change. During the on-site GLP determination, the pesticide-caused enzymatic AChE inhibition changed the platform’s colorimetric and fluorescence properties, thus providing the signal output in the LDCR of 0 to 10 μM with the LOD of 2.81 nM^154^ (Figure 6). A similar study developed a smartphone-dedicated kit test for selective GLP determination based on the Cu(II)-pyrocatechol violet complex. The kit sensed 20 μM GLP in tap water by the “naked eye” test, quantifying GLP with the LOD of 2.66 μM.^155^ Moreover, a novel enzyme-free visual ratiometric fluorescence paper sensor for GLP was devised by assembling blue CDs and AuNCs and incorporating them into a portable smartphone platform. GLP sensing relied on the quick (2 s) GLP-induced quenching of CD fluorescence originating from CD-GLP complex formation. The sensor allowed instant GLP determination in real samples with the exquisite LOD of 4.19 nM in the broad LDCR of 0–190 nM.^156^ Furthermore, in a similar study, a diverse (thiocholine-AuNPs)-based colorimetric smartphone-assisted sensor was devised to detect eight pesticides, including GLP, thiram, imidacloprid, tribenuron methyl, nicosulfuron, thiofensulfuron methyl, dichlorprop, and fenoprop. The modus operandi of the sensors relied on the pesticide-induced inhibition of AChE-mediated hydrolysis of the Au–S covalent bond in the thiocholine-AuNPs complex. This bond cleavage resulted in the RGB-valued color change associated with the alteration of the SPR properties of AuNPs. All pesticides were determined in real samples of fruits, vegetables, and traditional Chinese herbs, with the LOD below 0.15 μM, thus satisfying the established specification of the U.S. EPA, noted as ∼3.91 μM, and demonstrating the sensor’s applicability.^157^ Finally, a smartphone-integrated triple-mode SERS/fluorimetric/colorimetric sensor for GLP was devised based on flowerlike Zn MOFs and the HRP activity-mimicking H(2) L ligand. Unique properties of this ligand, including the enzymatic catalysis and Cu^2+^-sensitive fluorescence, were used to detect GLP in a florescence-quenching manner based on the Cu^2+^ chelation by GLP. This inhibiting interaction decreased both the catalytic activity of the H(2) L ligand and the optical and plasmonic properties of the nanocomposite, thus allowing for quantitative fluorescent/colorimetric/SERS determination of GLP with the LOD of 0.738 (0–0.12 μM LDCR), 2.26 nM (0–220 nM LDCR), and 0.186 nM (0.5–12 nM LDCR), respectively. Moreover, the sensor was integrated with a portable (test strips)-smartphone sensing platform dedicated to POC testing GLP in food samples.^158^

The extremely high technological advancement of the presented pioneer examples of selective and sensitive SM-based sensors sets the direction of action in analytical chemistry. Expectedly, the multimodal smartphone-based sensors will equip the agronomic and POC laboratories, providing automated, computerized, and high-throughput toxin determination, despite the relatively high production and maintenance costs. In the near future, smartphone-augmented sensors will be commonly used as components of nanobionic PW sensors. For example, PWs based on thin nanofilm electronics, such as SWCNTs field-effect transistors, can now detect dimethyl methylphosphonate, a volatile nerve agent, at the ppm level.^212^ Moreover, the flexible, stretchable, and adhesive PW sensor, fabricated by direct writing of chitosan-based inks, was active toward plant mechanical injury-responsive healing and sensed the intoxication with methyl parathion and nitrites.^213^

### 3.8 Robotic Devices for GLP Delivery

The GLP-resistant weeds’ evolution in the farmlands forced scientists and farmers to develop novel GLP-free technologies for modern weed management.^13,15^ Among the development of transgenic crops displaying resistance to auxinic herbicides and herbicides displaying inhibitory activity against acetolactate synthase, acetyl-CoA carboxylase, hydroxyphenylpyruvate dioxygenase or bioherbicides, and sprayable herbicidal ribonucleic acid interference (RNAi) agents, the nonbiochemical solutions have been considered as well.^13,214^ Aside from SM AI-enhanced sensors, these innovations involve soft terrestrial robotics that will give rise to robotic weeding in the nearest future.^13^ In 2022, the global agricultural robots market, including unmanned aerial vehicles (UAV), drones, automated harvesting system drones, and driverless tractors, was estimated to be worth 5.9 million USD and is anticipated to reach a forecast value of 30.5 million USD by 2032 (https://www.futuremarketinsights.com/reports/agriculture-robotics-market, access on 21.05.2023).

**Figure 7:**
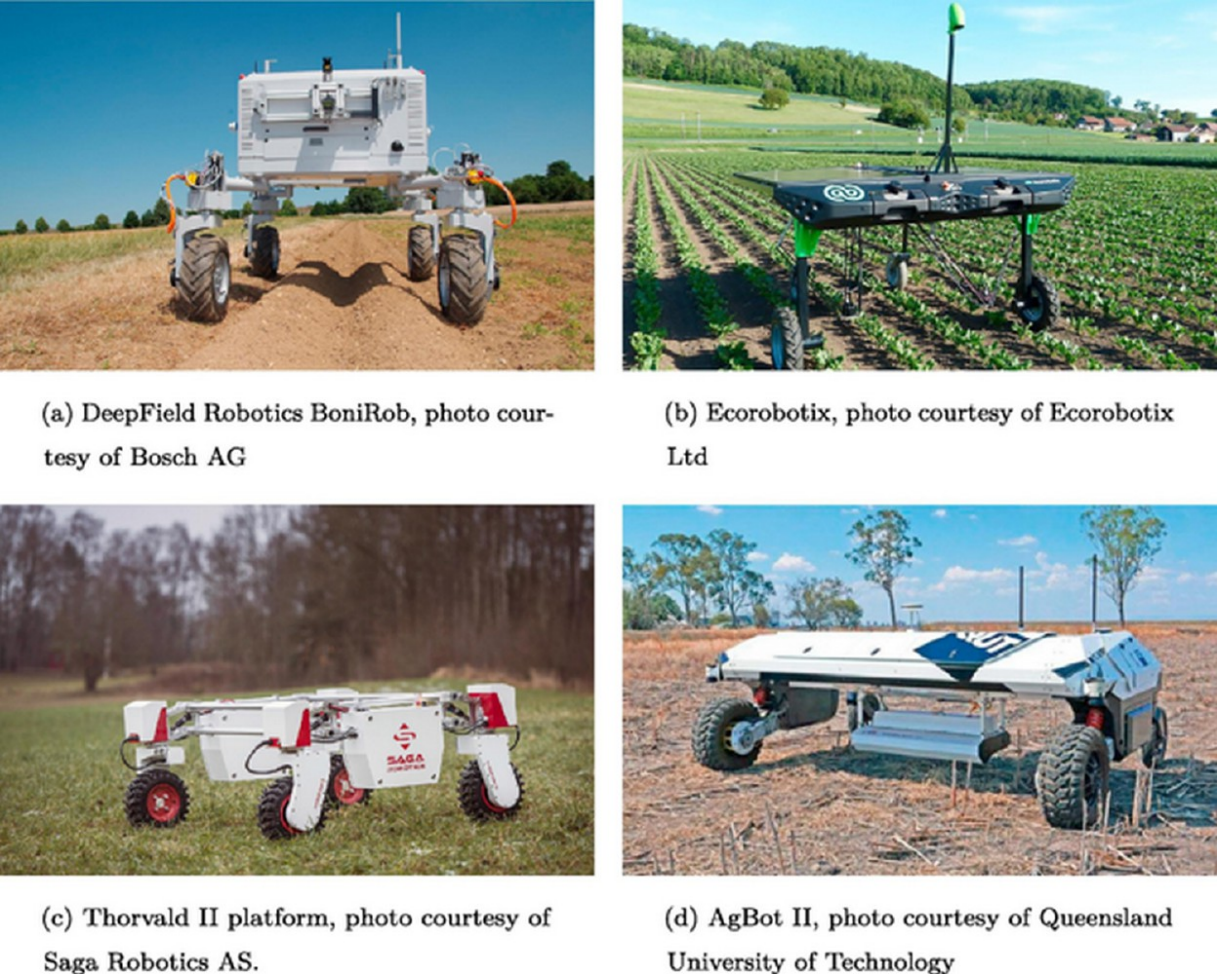
Robots used for in-row weed control, in-field detection, and site-targeted delivery of herbicides. Adapted with permission from ref (159). Copyright 2018 Elsevier.

Regarding GLP-oriented systems, a robot-based herbicide delivery system is a more advanced system for in-row weed control purposes. The robot was tailored to facilitate systematic and site-specific delivery of GLP and iodosulfuron droplets to four different weed species in a drop-on-demand manner without affecting carrot crops. The droplets were selectively sprayed on the leaves of the detected weeds within the plant row. Iodosulfuron, a sulfonylurea-based herbicidal inhibitor of acetolactate synthase, was used as an additive herbicide to control the species that GLP insufficiently controls. In indoor pot trials, amounts of 7.6 μg of GLP and 0.15 μg of iodosulfuron per droplet per plant were delivered, whereas in a field trial with the robot system involved, this amount was 5.3 μg of GLP per droplet. Moreover, the GLP amount was 10 times lower than GLP amount used in conventional crop spraying.^159^ In these in-field trials, the presented three-wheeled robot GLP delivery system displayed promising properties, including relatively low cost, maintainability, maneuverability, stability, and robustness, when compared to other commercial agricultural robots.^215,216^ (Figure 7). However, it would be required to perform quantitative environmental impact assessments using a comparative life cycle assessment of intra- and inter-row weeding management to claim its superior usefulness.^217^ Furthermore, worth mentioning the costs of devising and exploiting robotic GLP sensors/delivery systems, including costs of navigation hardware, machine vision technologies, and power consumption,^216^ are usually higher than those of conventional broadcast GLP analytical techniques, which must be considered when designing experiments or applying to industry. Not disregarding these cost limitations, in the long term, such robotic-based delivery systems may become a promising alternative for dosing a well-defined and strictly controlled amount of pesticides to the crops, thus reducing the risk of environmental contamination.

Robotics has become fundamental for constructing remote sensors (RSs) for GLP. They have been devised and implemented to monitor the agricultural and environmental quality of soil, crops, and water status. In particular, these tools have been applied to solve major issues of long-term herbicide-resistant weed management (HRWM).^36,37^ In the first step of modern HRWM, remote sensing is commonly implemented to detect and identify infested crops, followed by site-specific herbicide treatment of the weeds, mostly in their germination stage.^218^ Besides, RSs can be used for the man-free determination of herbicide-sprayed weeds and crops to eradicate the former or improve the latter’s growth using variable-rate technology (VRT).^219−224^

## 4 Future Prospective

Environment-protective farming (also called organic farming) is a goal of sustainable agriculture.^225^ Agriculture sustainability prevents soil degradation based on economic productivity, future crop yield maintenance, limited usage of agrochemicals, and eco-friendly integrated pest management and weed control. It ensures the health of the soil, air, water, livestock, animal, and humans.^226^ Concerning the latter, AI-excelled PA is expected to provide tools that enable rapid, mobile, and man-free delivery or detect harmful agrochemicals or biomaterials such as fertilizers and biocides. The AI technology aims to handle weed infestations because of their devastating role in crop cultivation by competing for water, nutrients, light, agricultural space, and air gases and by secreting potentially toxic exudates.^227,228^ Because weeds share the same zone and spread among crops, it is incredibly challenging to exterminate them without harming the crops. Besides, spectral images of herbicide-sprayed weeds and crops in early developmental stages are similar, which impedes proper visualization, recognition, and site-specific eradication.^229−231^ Nevertheless, AI-enhanced site-specific weed management, including weed patch mapping, will be possible.^20,232^ It shall allow for the application of nanoformulated herbicides in geospatially determined, stimuli-responsive, and dose-dependent manners, thus inhibiting weeds’ seed germination and decreasing weed biomass.^218,233^ Furthermore, tackling weed infestations can be addressed by UAV-handling of farmlands stricken with drought, air pollution, or heat caused by local climate changes that have been more frequent in the last ten years compared to previous decades.^234^

The limitation of the GBHs and agrochemical use was the founding myth of environment-protective farming, ecology, and weed science.^225^ Recent years’ technological advancement has displaced a previously in-force “many little hammers” approach based on the extensive labor and equipment-consuming field works.^235^ Nowadays, AI PA has been dominated by “digital farming,” providing the tool for intelligent and site-specific monitoring and improving crop protection. For instance, the controlled use of GBHs in recent years was associated with no-tillage agricultural systems, thus reducing their inevitable deficiencies, including soil degradation and high expenses. Thus, the current ban on GLP and GBH shall force farmers to return to conventional tillage systems as an effective weed management strategy for keeping crop-weed balance. So far, a series of no-tillage and no-GBH strategies have been developed to reduce the over-reliance on a few single modes of action herbicides, competitiveness in weeds, and dependency on herbicides, while enhancing the strategies of crop diversification, harvest weed seed control, and precision agriculture approaches, including RNAi technology, gene editing, robotics, and in-site or remote sensing.^235^

Expectedly, future devising of safe-by-design sensors for herbicides will mainly exploit AI tools of nanoinformatics,^236−239^ ML,^240−243^ and DL.^18,244−247^ Nanoinformatics-enhanced drug/sensor devising uses predictive risk assessment frameworks, including integrated approaches to testing and assessment for the in silico prediction, design, and optimization of the parameters of nanomaterials used as sensing components or delivery systems according to their agricultural destination.^18,248^ These parameters refer to structure and functionality as well as biocompatibility, stability, biodistribution, and transgenerational phytotoxicity of the material, thus providing prediction and translation of the long-term responses of the agroecosystem to current conditions, including efficient high or low doses of the agrochemical efficiently. This strategy is particularly promising because current experimental studies on nanomaterial toxicity require short-term exposures to relatively high doses of the agrochemical.^18^ Particularly, nanoinformatics models combine chemo- and bioinformatics tools with omics databases and in vitro, ex vivo, in vivo, and clinical data of the active agents and target or nontarget species, allowing for the de novo design of ActI or nanomaterials.^237^ The integrated web repositories and database were originally created using the data of eight model plant species, i.e., *Arabidopsis thaliana*, *Oryza sativa*, *Solanum lycopersicum*, *Sorghum bicolor*, *Vitis vinifera*, *Solanum tuberosum*, *Medicago truncatula*, and *Glycine max*. They enabled the founding of interspecies and intermolecular interaction networks such as Plant Omics Center, Plant Secretome, and Subcellular Proteome KnowledgeBase. Associated with analogic databases concerning microbiota, agrochemicals (PEST-CHEMGRIDS, Pesticide Properties Database, Pesticide Target Interaction Database, PubChem), hydroclimatic indicators, and socioeconomic and anthropogenic inputs, these nanoinformatics tools facilitate comprehensive and predictive determination of molecules, species, and plant diseases at every possible level.^37,249−251^ Regarding the nanoinformatics-based GLP sensing, the site-targeted performance of the future smart sensor shall be applied to the GLP-sprayed areas to determine GLP in the chemical environment of natural species.

The ML and DL methods have already been applied to crop sensing and phenotyping at all scales, including lands, fields, canopies, and leaves.^240−243^ Thus, presumably, these powerful computing tools, incorporated in the future smart sensors, will be easily harnessed to predict tripartite (e.g., “agrochemical-soil-plant,” “pathogen-treatment-plant”) interactions under variable climate conditions.^18,244−247^ The DL-enhanced algorithms will process the object and spectral data regarding the images’ numbers, types, sizes, and resolutions by grouping proximal pixels (of a few millimeters or centimeters) with homogeneous spectral value, combining them spectrally, topologically, and contextually.^252^ Upon spatiotemporal evaluation of the vegetation indices, including temperature, biomass, moisture, weed infestation, and nitrogen balance index (chlorophyll-to-polyphenol ratio) of these objects, the smart sensors shall identify the nature of the stress and apply proper fertilizers, agrochemicals, and irrigation waters in a site-specific manner using VRT-based equipment.^253^ In effect, the computational tool-excelled smart sensors will acquire and process massive amounts of data on “what,” “how,” “where,” and “when” the human intervention should be applied to address the most pressing needs, thus reducing the consumption of herbicides, such as GLP, to an absolute minimum.^244−247^

## List of abbreviations

ACh: Acetylcholine
AChE: Acetylcholinesterase
ActI: Active ingredient
AFM: Atomic force microscopy
AgNC: Silver nanocluster
AgNP: Silver nanoparticle
AHS: Agricultural Health Study
AI: Artificial intelligence
AMPA: (Aminomethyl) phosphonic acid
ATU: 1-Allyl-2-thiourea
AuNC: Gold nanocluster
AuNP: Gold nanoparticle
BF: Binding factor
CD: Carbon dot
CNT: Carbon nanotube
COVID-19: Coronavirus disease
CTAB: Cetyltrimethylammonium bromide
CV: Cyclic voltammetry
DA: Dopamine
DAD: Diode array detector
DHEBA: *N*,*N*′-(1,2-Dihydroxyethylene) bis(acrylamide)
DHP: Direct hemoperfusion
DL: Deep learning
DMAEM: 2-Dimethyl aminoethyl methacrylate
DNA: Deoxyribonucleic acid
DPPC-Cl: 3,6-Dimethoxy-9-phenyl-9*H*-carbazole-1-sulfonyl chloride
ECETOC: European Centre for Ecotoxicology and Toxicology of Chemicals
EChA: European Chemical Agency
ECL: Electrochemiluminescence
EFSA: European Food Safety Authority
EGDMA: Ethylene glycol dimethacrylate
EIS: Electrochemical impedance spectroscopy
ELISA: Enzyme-linked immunosorbent assay
ENM: Engineered nanomaterial
EPA: Environmental Protection Agency
EPSPS: 5-Enolpyruvynyl-shikimate-3-phosphate synthase
ESI-MS: Electrospray ionization mass spectrometry
FAO: Food and Agriculture Organization
FLD: Fluorescence detector
FMOC: Fluorenylmethyloxycarbonyl (derivative)
FRET: Förster resonance energy transfer
FRP: Free radical polymerization
GBH: Glyphosate-based herbicide
GC: Gas chromatography
GCE: Glassy carbon electrode
GC-EIMS: Gas chromatography coupled to electron ionization mass spectrometry
GE: Genetically engineered (organism)
GLP: Glyphosate
GLP-IPA: Glyphosate isopropylamine
GLP-SH: Glyphosate surfactant
GMO: Genetically modified organism
GO: Graphene oxide
HPLC: High-performance liquid chromatography
HRMS: High-resolution mass spectrometry
HRP: Horseradish peroxidase
HRWM: Herbicide-resistant weed management
ICP-MS: Inductively coupled plasma mass spectrometry
Ig: Immunoglobulin
IPA: Isopropylamine
IRMS: Isotope-ratio mass spectrometry
ISPAg: International Society of Precision Agriculture
LC: Liquid chromatography
LC–IRMS: Liquid chromatography coupled with isotope ratio mass spectrometry
LDCR: Linear dynamic concentration range
LOC: Lab-on-a-chip
LOD: Limit of detection
LOQ: Limit of quantification
MAA: Methacrylic acid
MBA: *N*,*N*′-Methylenebis(acrylamide)
MIP: Molecularly imprinted polymer
ML: Machine learning
MOF: Metal–organic framework
MS: Mass spectrometry
MWCNT: Multiwalled carbon nanotube
NC: Nanocluster
NFC: Near-field communication
NIR: Near-infrared (light)
NMDA: *N*-Methyl-d-aspartate
NT: Nanotube
OP: Organophosphate
OVA: Ovalbumin
PA: Precision agriculture
PAA: Polyacrylic acid
μPAD: Microfluidic paper-based analytical device
PANI: Polyaniline
PCR: Polymerase chain reaction
PDA: Polydopamine
PEG: Poly(ethylene) glycol
PNIPAM: Poly(*N*-isopropylacrylamide)
POC: Point-of-care
POEA: Polyoxyethyleneamine
PPy: Polypyrrole
PVC: Poly(vinyl chloride)
PW: Plant wearable
QCM: Quartz crystal microbalance
QD: Quantum dot
RGB: Red-green-blue (analysis)
rGO: Reduced graphene oxide
RNAi: Ribonucleic acid interference
RS: Remote sensor
SCP: Soft colloidal particle
SDS-PAGE: (Sodium dodecyl sulfate)-(polyacrylamide gel electrophoresis)
SERS: Surface-enhanced Raman spectroscopy
SiNP: Silica nanoparticle
SM: Smart material
SPCE: Screen-printed carbon electrode
SPE: Solid-phase extraction
SPR: Surface plasmon resonance
SWCNT: Single-walled carbon nanotube
TLC: Thin-layer chromatography
UAV: Unmanned aerial vehicle
UPLC: Ultraperformance liquid chromatography
UPLC-Q-ToF-HRMS/MS: Ultraperformance liquid chromatography coupled with quadrupole-time-of-flight mass spectrometry
UV: Ultraviolet (light)
VRT: Variable-rate technology
WHO: World Health Organization
WSN: Wireless sensor network
ZSM: Zeolite Socony Mobil
